# Alternatives to vitamin B_1_ uptake revealed with discovery of riboswitches in multiple marine eukaryotic lineages

**DOI:** 10.1038/ismej.2014.146

**Published:** 2014-08-29

**Authors:** Darcy McRose, Jian Guo, Adam Monier, Sebastian Sudek, Susanne Wilken, Shuangchun Yan, Thomas Mock, John M Archibald, Tadhg P Begley, Adrian Reyes-Prieto, Alexandra Z Worden

**Affiliations:** 1 Monterey Bay Aquarium Research Institute, Moss Landing, CA, USA; 2 School of Environmental Sciences, University of East Anglia, Norwich, UK; 3 Department of Biochemistry and Molecular Biology, Dalhousie University, Halifax, Nova Scotia, Canada; 4 Integrated Microbial Biodiversity Program, Canadian Institute for Advanced Research, Toronto, Ontario, Canada; 5 Department of Chemistry, Texas A&M University, College Station, TX, USA; 6 Biology Department, University of New Brunswick, Fredericton, New Brunswick, Canada

## Abstract

Vitamin B_1_ (thiamine pyrophosphate, TPP) is essential to all life but scarce in ocean surface waters. In many bacteria and a few eukaryotic groups thiamine biosynthesis genes are controlled by metabolite-sensing mRNA-based gene regulators known as riboswitches. Using available genome sequences and transcriptomes generated from ecologically important marine phytoplankton, we identified 31 new eukaryotic riboswitches. These were found in alveolate, cryptophyte, haptophyte and rhizarian phytoplankton as well as taxa from two lineages previously known to have riboswitches (green algae and stramenopiles). The predicted secondary structures bear hallmarks of TPP-sensing riboswitches. Surprisingly, most of the identified riboswitches are affiliated with genes of unknown function, rather than characterized thiamine biosynthesis genes. Using qPCR and growth experiments involving two prasinophyte algae, we show that expression of these genes increases significantly under vitamin B_1_-deplete conditions relative to controls. Pathway analyses show that several algae harboring the uncharacterized genes lack one or more enzymes in the known TPP biosynthesis pathway. We demonstrate that one such alga, the major primary producer *Emiliania huxleyi*, grows on 4-amino-5-hydroxymethyl-2-methylpyrimidine (a thiamine precursor moiety) alone, although long thought dependent on exogenous sources of thiamine. Thus, overall, we have identified riboswitches in major eukaryotic lineages not known to undergo this form of gene regulation. In these phytoplankton groups, riboswitches are often affiliated with widespread thiamine-responsive genes with as yet uncertain roles in TPP pathways. Further, taxa with ‘incomplete' TPP biosynthesis pathways do not necessarily require exogenous vitamin B_1_, making vitamin control of phytoplankton blooms more complex than the current paradigm suggests.

## Introduction

**Figure 1:**
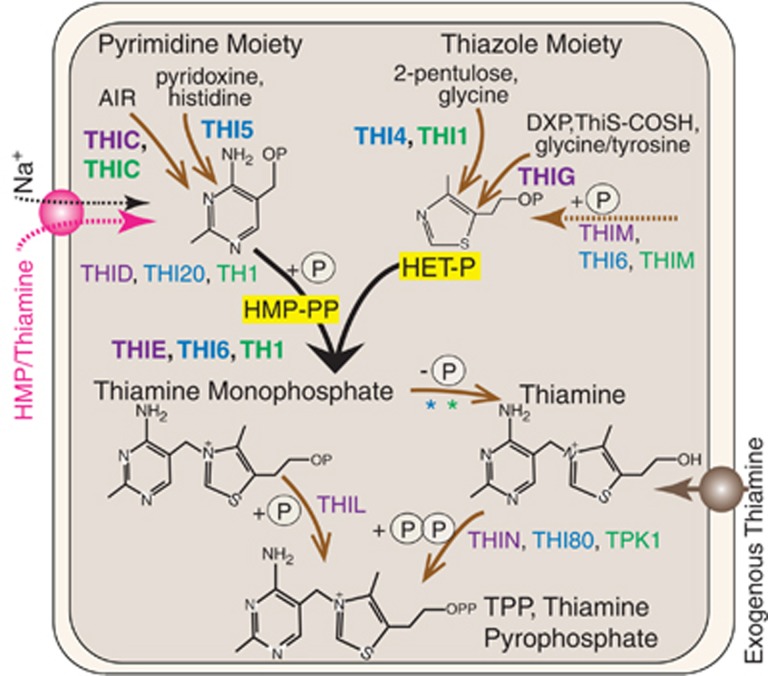
The TPP biosynthesis pathway in plants (green), bacteria (purple) and fungi (blue). Arrows indicate different routes taken to the following product (brown) or the universal precursor condensation step (black). The pyrimidine moiety (HMP-P) is synthesized using THIC (plants and bacteria) or THI5 (fungi). In plants and fungi, the thiazole moiety (HET-P) is synthesized by homologous THI1 and THI4, respectively. Bacteria (and diatoms) synthesize HET-P (as HET with a phosphate) using a number of enzymes, including THIG. TMP is universally formed by condensation of HMP-PP and HET-P via the TMP synthase portion (THIE) of the bifunctional enzymes TH1 (plants and bacteria; THID, HMP-P kinase and THIE) or THI6 (fungi; THIE and THIM, HET kinase). In some bacteria, *THIE* is found as a single gene. Relevant kinases and non-specific hydrolases (indicated by ‘*') are depicted and discussed in detail along with pathway chemistry in Jurgenson *et al.* (2009). Pink represents an as yet unidentified HMP or HMP/thiamine transporter or symporter (as depicted) in *E. huxleyi* and other algae (SSSP and SSSQ are hypothesized candidate transporters discussed herein). AIR, 5-aminoimidazole ribonucleotide; DXP, 1-deoxy-D-xylulose-5-phosphate; THIS-COSH, THIS-thiocarboxylate; HMP, 4-amino-5-hydroxymethyl-2-methylpyrimidine; HET-P, 4-methyl-5-β-hydroxyethylthiazole phosphate or, in yeasts, 4-methyl-5-β-hydroxyethylthiazole adenosine diphosphate, abbreviated HET-P here for simplicity; P, a phosphate group.

Thiamine pyrophosphate (TPP)—the biologically active form of vitamin B_1_—is vital for all cellular life because it is a co-factor for several essential enzymes (Jurgenson *et al.,* 2009). Oceans have long been thought vitamin B_1_ (B_1_) deplete and studies have now demonstrated that thiamine can be absent or below picomolar detection limits in marine surface waters (Sanudo-Wilhelmy *et al.*, 2012). Nevertheless, many eukaryotic algae that contribute significantly to primary production, as well as harmful algal bloom species, reportedly rely on exogenous B_1_ (Croft *et al.,* 2006; Tang *et al.,* 2010). For example, the haptophyte *Emiliania huxleyi*, which forms massive blooms that can be detected from space, is considered a thiamine auxotroph (unable to synthesize B_1_) (Bertrand and Allen, 2012; Read *et al.,* 2013). In contrast, fungi, plants, diatoms and many bacteria are capable of *de novo* B_1_ synthesis (Croft *et al.,* 2006; Cheah *et al.,* 2007; Jurgenson *et al.,* 2009). These thiamine prototrophs produce two precursor moieties that are universally condensed to thiamine monophosphate (TMP), before phosphorylation to TPP (Figure 1) (Jurgenson *et al.,* 2009). However, the enzymes and substrates used to produce the pyrimidine (4-amino-5-hydroxymethyl-2-methylpyrimidine phosphate, HMP-P) and thiazole (4-methyl-5-β-hydroxyethylthiazole phosphate or 4-methyl-5-β-hydroxyethylthiazole adenosine diphosphate, HET-P) precursor moieties differ between lineages.

In the marine diatoms *Thalassiosira pseudonana* and *Phaeodactylum tricornutum*, as well as chlorophyte algae, plants, and some fungi, genes encoding enzymes responsible for HMP-P synthesis are controlled by TPP riboswitches (Croft *et al.*, 2007; Wachter, 2010). This is also the case for genes encoding HET-P synthesis enzymes in some chlorophyte algae, non-vascular plants and fungi. Riboswitches are a specialized form of mRNA-based gene regulation used extensively by bacteria, typically for feedback regulation of biosynthesis or transport of a particular metabolite bound by the riboswitch aptamer domain (Wachter, 2010; Breaker, 2011). In bacteria, entire thiamine biosynthesis operons can be under TPP riboswitch control (Breaker, 2011). The riboswitch binding interaction is highly specific for the target small molecule and causes a conformational change that results in various forms of gene control without involvement of other (regulatory) proteins. Mechanisms of control include suppressing expression, eliciting expression, initiating or disrupting translation, and, in eukaryotes, inducing alternative splicing (Winkler and Breaker, 2005; Cheah *et al.,* 2007; Wachter, 2010; Breaker, 2011).

Only the riboswitch class responding to TPP has been found in eukaryotes, although bacteria have numerous classes (Wachter, 2010; Breaker, 2011). In plants, control of functionally related genes without the involvement of additional proteins is thought to reduce response rates and energy costs associated with thiamine biosynthesis (Bocobza and Aharoni, 2008). This efficient form of regulation would seem particularly advantageous for eukaryotic phytoplankton in marine systems where B_1_ and nutrients are often at low concentrations (Sanudo-Wilhelmy *et al.*, 2012; Carini *et al.,* 2014). Yet only some green algae and two diatoms are known to have TPP riboswitches (Cheah *et al.,* 2007; Croft *et al.*, 2007; Worden *et al.,* 2009; Wachter, 2010).

Here, we sequenced transcriptomes from ecologically important marine green algae (prasinophytes) and analyzed these alongside published algal genome sequences. We discovered TPP riboswitches are present in many eukaryotic phytoplankton, but are affiliated with genes that do not have known roles in TPP biosynthesis. These results led us to conduct genome surveys to examine potential thiamine prototrophy or auxotrophy for taxa harboring the novel riboswitch-containing genes. Growth experiments conducted using a subset of these phytoplankton demonstrated that the novel genes are highly expressed under thiamine deprivation. We therefore developed the hypothesis that several marine phytoplankton with B_1_ biosynthesis pathway gaps are capable of growth using alternative pathway components. The hypothesis was tested in *E. huxleyi*, which we demonstrate grows equally well on HMP or thiamine. The results open new research directions for understanding the function of several uncharacterized but thiamine-responsive genes, their control by riboswitches, and thiamine's role in regulating marine microbial growth. Collectively, our studies emphasize that the paradigm of exogenous B_1_ being a major phytoplankton control should be reexamined.

## Materials and methods

### Riboswitch identification and characteristics of associated genes

Riboswitches were initially detected in *Micromonas* sp. RCC299-to-*Micromonas pusilla* CCMP1545 genome alignments based on high conservation in non-coding regions. Twenty-three eukaryotic algal genomes and four algal transcriptomes generated here (see below, Supplementary Table S1) were searched using BLASTP and TBLASTN for proteins encoded by the novel *Micromonas* genes with associated riboswitches, *SSSF*, *SSSP*, *ATS1* and *UNK1*. The retrieved sequences and genes encoding known TPP biosynthesis enzymes (see below) were screened for the universally conserved ‘cugaga' TPP-riboswitch motif (Wachter *et al.,* 2007) throughout coding domain sequences, introns and untranslated regions (UTRs) or, in the absence of UTRs, 500 bp upstream and downstream of coding domain sequences. When found, P5 pairing and the resulting loop were searched for, and, if present, secondary structures were further resolved by manually testing base pairing possibilities and loop formation until structure completion. Detected riboswitch sequences were used as BLASTN queries against the resident genome. Putative homologs recovered from non-prasinophyte algae were also used as queries in additional BLASTP searches (against other algae). We also searched non-photosynthetic stramenopile genomes as well as the *Perkinsus marinus* genome and a *Karenia brevis* transcriptome (http://camera.calit2.net/mmetsp/details.php?id=MMETSP0030) because a complete photosynthetic alveolate genome sequence was not available. Negative results are not considered conclusive because P3 length differences, inconsistent P3a presence, and limited overall nucleotide conservation make riboswitch stem pairing difficult to identify. Transmembrane helix predictions were performed on SSSF, SSSP, SSSQ, ATS1 and UNK1 using HMMTOP v. 2.0 (Tusnády and Simon, 1998).

### Transcriptome preparation and sequencing

*Pyramimonas parkeae* CCMP725, *Pyramimonas amylifera* CCMP720, *Micromonas* NEPCC29 and *Micromonas* CCMP2099 were grown on a 14:10-h light:dark cycle (80 μmol photon m^−2^ s^−1^ photosynthetically active radiation). Cells were cultured in f/2 (CCMP725, CCMP720) or K (NEPCC29, CCMP2099) media (Anderson, 2005) at 20–21 °C or 6 °C (CCMP2099 only). Cultures were monitored by fluorometry or flow cytometry and harvested after ⩾3 generations of exponential growth. 18S rRNA genes were sequenced using methods in Worden *et al.* (2004) to verify cultures were mono-algal. RNA was harvested 2 h before and after lights-on and extracted using the TotallyRNA kit (Life Technologies, Carlsbad, CA, USA) with bead beating (1 min; 200 μl glass beads and 1 ml lysis buffer). Genomic DNA was removed using the TurboDNA-free kit (Life Technologies). Pre- and post-dawn RNA was combined equally before sequencing. Transcriptome assembly was performed on polyA selected pair-ended Illumina sequences using Batch Parallel Assembly v1.0 (Keeling *et al.*, 2014). 18S and 16S rRNA contigs were blasted against NCBI's nr nucleotide database and verified as matching the expected species; the *P. parkeae* transcriptome contained some stramenopile sequences, therefore best identity to *P. amylifera* (versus other taxa) was used to verify the origin of sequences presented here.

### Searches for known thiamine biosynthesis-related proteins and transporters

A query set of ‘core' thiamine biosynthetic proteins (THIC, THI5, THIG, THI4, THIE, TH1, THI6, THIM and THID) from model plants, fungi, and bacteria was used in BLASTP and TBLASTN searches against taxa in which riboswitches were identified (Supplementary Table S2). Searches using Pfam models (Finn *et al.,* 2010) were also performed and candidate proteins analyzed further when *E*-values were ⩽1e^−15^. Acquired sequences were used as queries in iterative BLASTs. Recovered THIG sequences were designated as bacterial-like. Distinctions between plant-like (THI1) and fungal-like (THI4) homologs (as well as THIC) were based on taxonomic composition of BLASTP hits (with cutoffs ⩾50% query coverage and *E*-value ⩽10^−50^) in NCBI nr. To avoid biases due to uneven taxon distributions, we also performed searches in nr with the most closely related group excluded. Members of Pfam PF09084 detected in algae (Supplementary Table S3) were not assigned putative functional roles given their equivalent distances to bacterial aliphatic sulfonate ABC transporters (SSUA) and THI5 members of this Pfam. Additionally, *E. huxleyi* (containing two SSUA/THI5-like proteins) has a complete thiamine pathway except THIC, and is a thiamine auxotroph (Bertrand and Allen, 2012) making it unlikely algal SSUA/THI5-like proteins are directly involved in HMP-P synthesis. Using the above methods we also searched for homologs of the bacterial ABC thiamine transporter components THIT, THIB, THIP and THIQ (Webb *et al.,* 1998; Erkens and Slotboom, 2010) and the high affinity thiamine transporter THI10 as well as THI72, THI73 and PHO3 from yeast (Nosaka, 2006).

### Thiamine manipulation experiments

Phytoplankton were grown at 21 °C in media made with an artificial seawater base (see http://www.mbari.org/phyto-genome/pdfs/KASW.pdf) and monitored daily by flow cytometry. Thiamine HCl and 4-methyl-5-β-hydroxyethylthiazole were purchased (Sigma-Aldrich, St. Louis, MO, USA; T4625 and W320404, respectively). 4-amino-5-hydroxymethyl-2-methylpyrimidine was synthesized according to Reddick *et al.* (2001). Precursor and water purity was tested by plating *E. coli* mutants lacking *THIC*, *THIG* or *THIE* on media prepared with 1 μM HMP (but no-thiamine or HET); 1 μM HET (but no HMP or thiamine); and 1 μM thiamine (but no HET or HMP). These deletion mutants showed growth/no-growth patterns expected only if the precursors and water used for media preparation were thiamine-free.

Axenic *Micromonas* sp. RCC299 and axenic *Micromonas pusilla* CCMP1545 were acclimated to thiamine replete medium under a 13:11 or 14:10-h light:dark cycle (∼130 μmol photon m^−2^ s^−1^ photosynthetically active radiation) and maintained in mid-exponential growth for ⩾10 generations before each experiment. Each species was grown in its ‘best performing' medium: KASW (RCC299) and L1ASW (CCMP1545) (Anderson, 2005). Pilot studies using 0.3 (control), 1, 10 and 100 μM thiamine concentrations showed growth inhibition for CCMP1545 (but not RCC299) at 100 μM. Hence, 1 μM (CCMP1545) and 10 μM (RCC299) were used for high thiamine and precursor treatments.

At experiment start, *Micromonas* cultures were centrifuged twice to remove cells from thiamine-containing medium. Experimental treatments were performed with three or four biological replicates. The experiments included negative controls (no-thiamine) and no-thiamine treatments supplemented with HMP, HET or both, as well as a high thiamine treatment and positive controls. RCC299 was inoculated at 3 810 000±253 000 cells ml^−1^, and grown without further manipulation (‘follow-out' experiment) or, transferred regularly in a ‘transfer' experiment comparing growth in negative (no-thiamine) and positive controls. CCMP1545 was inoculated at 4 150 000±180 000 cells ml^−1^ and transferred regularly. Cells in transfer experiments were diluted to their starting concentration every 24 h (unless the treatment growth rate reached zero) to avoid limitation by other medium constituents. For RNA sampling, cells were centrifuged and pellets frozen at −80 °C.

Axenic *E. huxleyi* CCMP2090 was purchased (NCMA, Bigelow, ME, USA) while *E. huxleyi* CCMP1516 was provided by B. Read. Both were grown in L1ASW on a 14:10-h light:dark cycle (200 μmol photon m^−2^ s^−1^ photosynthetically active radiation). Exponentially growing cells were inoculated into no-thiamine medium (1:25 thiamine replete culture:medium) at 176 000±4000 (CCMP2090) or 131 000±7782 (CCMP1516) cells ml^−1^ at experiment start. Thiamine or precursor moieties were added at 1 μM (final concentration) to achieve various treatments. To maintain medium constituents at ‘replete' levels, cells were transferred regularly into fresh medium throughout the experiments. Cells were not transferred lower than their starting concentrations to avoid induction of lag phase, making the frequency of transfer treatment dependent. Additionally, at day 6, a series of CCMP2090 cells from each treatment (and controls) were no longer transferred but instead monitored until experiment termination.

During experiments, cultures were inoculated into organic rich test medium (5 g peptone plus 10 g malt extract in 1 l seawater) and incubated in the dark at 21 °C. Cultures were also observed by DAPI staining with visualization using epifluorescence microscopy (Porter and Feig, 1980). Axenicity was verified for CCMP2090, CCMP1545 and RCC299.

### QPCR

RNA was extracted using the RNeasy Mini kit (Qiagen, Valencia, CA, USA) according to the manufacturer's instructions and gDNA removed using the TurboDNA-free kit (Life Technologies). RNA was quantified, diluted to a uniform concentration, and reverse transcribed using the Superscript III First Strand Synthesis System (Life Technologies) and oligo-dT primers. Minus-RT reactions were also performed. Linear dynamics curves were analyzed to ensure linear RNA to cDNA conversion, resulting in subsequent use of 1 ng μl^−1^ (final concentration).

QPCR TaqMan primer/probe sets were designed using the *Micromonas* genomes/gene models (Supplementary Table S4). Plasmids representing genes of interest were quantified spectrophotometrically, and diluted to create standards ranging from 10^8^ to 10^1^ copies rxn^−1^, in 10-fold increments for primer efficiency tests. Reaction efficiencies were calculated as (10^(−1/*m*)^-1), where *m* is the slope of the regression line and ranged from 95% to 109%. QPCR was performed on an AB7500 in 25 μl volumes using 12.5 μl Gene Expression Master Mix (Life Technologies), 2.25 μl of each primer (900 nM), 0.63 μl probe (250 nM) and either 1 ng cDNA rxn^−1^ or plasmid standards, under the conditions: 50 °C, 2 min; 95 °C, 10 min; followed by 40 cycles of 95 °C, 15 s and 60 °C, 1 min. Samples, no-template and positive controls were run in triplicate. Raw fluorescence was normalized using a 0.2 threshold and 3–15 cycle baseline. Outliers were removed using Dixon's q-test (95% cutoff) (Dean and Dixon, 1951). C_T_'s >36 were excluded. Fold changes were calculated using the delta delta C_T_ method (Livak and Schmittgen, 2001), with *Beta-tubulin* and *Actin* as housekeepers, and positive controls as calibrators. Final analyses used *Beta-tubulin* due to its lower overall variation. Fold changes were averaged over technical triplicates and standard deviations calculated for biological replicates. R (v2.1.13, www.r-project.org) was used for statistical analyses.

### Phylogenetic reconstructions

**Figure 2:**
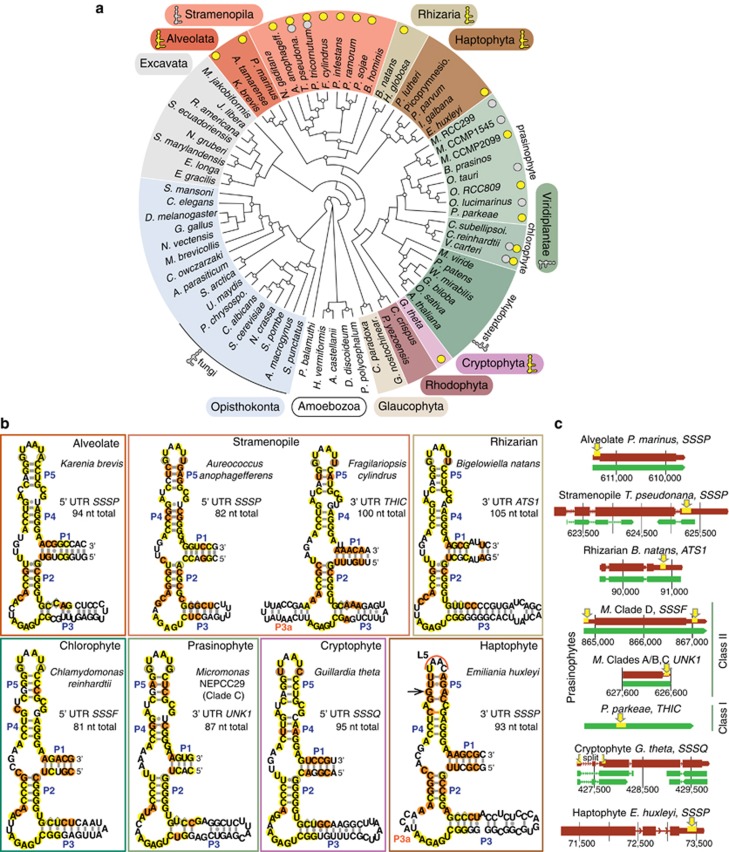
Newly discovered riboswitches span eukaryotic supergroups. (**a**) Icons (major lineage level) and circles (individual species) indicate the presence of newly identified (yellow) or previously known (gray) riboswitch(es). Numbers of riboswitches found, or previously known, and characteristics of the riboswitch and affiliated gene are given in Supplementary Table S2. Streptophyte and fungal species were not searched and species with riboswitches are not depicted because uniform information is lacking for these groups. Bootstrap support ⩾70% is shown (white circles) for this representative cladogram and the tree is rooted for display purposes only. (**b**) Predicted secondary structures for 8 of 31 riboswitches identified here (for others, see Supplementary Figure S1). Circles indicate nucleobases and pairing conserved in plants, that are identical (yellow) or divergent from plants (orange) in the unicellular eukaryotes studied. Plant positions that are conserved, but can be A or G (outlined circles), or C or U (outlined diamonds), are also indicated. The maximum total length depicted is 105 nt but can be up to 183 nt (Supplementary Table S2). (**c**) Riboswitch positioning (yellow rectangles emphasized by arrows) in a selection of predicted gene models (rust) is shown with bars representing exons (thick), UTRs (intermediate width), introns (thin) and expression data (green) from ESTs or RNA-seq. Numbers represent genome scaffold positions.

A concatenated alignment of 16 conserved nucleus-encoded proteins (5679 total positions) adapted from Parfrey *et al.* (2010) and Read *et al.* (2013) was used to infer eukaryotic relationships (Figure 2a). Additional prasinophyte and stramenopile sequences were incorporated using MAFFT v7 (Katoh and Standley, 2013) ‘add' option; G-INS-1 and the BLOSUM62 scoring matrix. For SSSP and SSSQ, three iterations of PSI-BLAST were used in the NCBI-nr database and genome portals (Supplementary Table S1) to avoid query-biased searches and to detect remote homologs. Sequences with an *E*-value of <1e^−20^ were used to build a position-specific score matrix, used for subsequent PSI-BLAST iterations. Protein sequences were aligned using MAFFT v7 with slow/global homology parameters (G-NS-i mode) with BLOSUM45 scoring matrix and alignments manually curated. Each best maximum-likelihood phylogenetic tree was identified from 25 reconstructions computed using RAxML v7.5 (Stamatakis, 2006) with model selection (LG+G+F) by ModelGenerator v0.85 (Keane *et al.,* 2006) and AIC criterion. In all, 100 bootstrap replicates were computed. Eukaryotic, bacterial and archaeal sequences in the supported clade containing algal SSSP/SSSQ homologs were extracted and re-aligned to maximize available positions and analyzed using maximum-likelihood methods as above. Posterior probabilities were computed with MrBayes v3.2.1 (Ronquist and Huelsenbeck, 2003), using two runs of four MCMC chains across 1 000 000 generations, of which the first quarter was discarded (burn-in).

## Results

### Discovery of riboswitches in multiple phytoplankton lineages

We identified putative riboswitches in 15 of 23 publically available eukaryotic algal genome sequences, including *Guillardia theta* (cryptophytes), *E. huxleyi* (haptophytes), *Bigelowiella natans* (rhizaria) and *Aureococcus anophagefferens* (stramenopiles) (Figure 2a; Supplementary Table S2). New riboswitches were also detected in genomes from diatoms, Class II (Mamiellophyceae) prasinophytes, and chlorophytes (Figure 2a), but searches of trebouxiophytes, rhodophytes, and a glaucophyte returned negative results. We also identified riboswitches in heterotrophic groups that may have photosynthetic ancestors (Keeling, 2010), specifically oomycetes (*Phytophthora* species), the marine labyrinthulomycete *Aplanochytrium kerguelensis*, and the marine alveolate *Perkinsus marinus*. To expand taxonomic resolution for marine phytoplankton, we sequenced transcriptomes from Class I (*P. parkeae* and *P. amylifera*) and additional Class II prasinophytes (*Micromonas* spp. CCMP2099 and NEPCC29). Searches of these and an available *K. brevis* (dinoflagellate) transcriptome rendered additional riboswitches (Supplementary Table S2). The 31 putative riboswitches identified are affiliated with eight different genes.

Six of the putative riboswitches are associated with TPP biosynthesis-related genes (Supplementary Table S2). In *G. theta*, we identified a riboswitch affiliated with *THIM* (HET kinase). In *Micromonas* RCC299 and NEPCC29 as well as *A. anophagefferens*, riboswitches are associated with *SSUA/THI5*-like genes. These encode members of Pfam PF09084 that are distant from other Pfam members, specifically SSUA (bacterial aliphatic sulfonate ABC transporters) and THI5 (Figure 1) proteins, precluding functional assignment. We also identified riboswitches in *THIC* genes from the diatoms *Fragilariopsis cylindrus* and *Pseudo-nitzschia multiseries* as well as Class I prasinophytes (Supplementary Table S2).

Twenty-five of the newly identified eukaryotic riboswitches are associated with poorly characterized genes. Proteins encoded by three novel riboswitch-containing genes (referred to here as *SSSF*, *SSSP* and *SSSQ*) belong to Pfam PF00474, a sodium:solute symporter family that contains transporters of diverse metabolites, nucleobases and vitamins. Fifteen transmembrane helices are predicted for SSSF, whereas thirteen are predicted for both SSSP and SSSQ. Potential functions for proteins encoded by the other two novel riboswitch-containing genes are less clear. Both lack motifs and multiple predicted transmembrane helices that would be suggestive of transporter roles. Algal Thiamine Sensitive 1 (named here, ATS1) has sequence similarity to the folate receptor family (PF03024), exclusively eukaryotic proteins thought to bind folate and reduced folic acid. The protein Unknown 1 (named here, UNK1) does not have significant similarity to known protein domains.

### Characteristics of newly discovered riboswitches and positioning within genes

Most of the predicted riboswitches share secondary structure with characterized plant TPP riboswitches (Figure 2b; Supplementary Figure S1). They also share specific nucleotide pairing involved in P2, P4 and P5 stem formation in plants. P3 stem length is relatively consistent within any individual species, but ranges from 7 to 115 bases across taxa investigated (Supplementary Table S2). In some diatom riboswitches, we observed an additional stem region (P3a, Supplementary Figure S1a), common in bacteria (Wachter *et al.,* 2007). Moreover, in the *E. huxleyi* riboswitch, a conserved P5 stem pair is absent (black arrow, Figure 2b) and the L5 loop is bacterial-like.

The location of riboswitches relative to coding regions of their associated genes is variable (Figure 2c; Supplementary Table S2). Among prasinophytes, we found riboswitches mid-gene, or in UTRs at either end of genes. For example, in *Micromonas* Clade A/B (RCC299), C (NEPCC29) and E (CCMP2099) riboswitches are in 3′ UTRs while in *Micromonas* Clade D (CCMP1545) they are present in both 5′ and 3′ UTRs of the same gene(s), and in all *Ostreococcus* they are in 5′ UTRs. These findings extend to other lineages such as stramenopiles, where *A. anophagefferens* has riboswitches in UTRs at opposite ends of different genes.

Additionally, the relative positioning of some newly identified riboswitches and architecture of the associated genes is different from those observed in model taxa. At least 14 of these genes do not appear to undergo splicing (Supplementary Table S2). The *A. anophagefferens* and *Micromonas* sp. RCC299 *SSUA/THI5*-like genes with 3′ UTR riboswitches are single exon genes with no introns. Likewise, most marine algal *SSSP* and *SSSF* homologs are in 5′ UTRs of single exon genes without apparent spliceoforms. The three 5′ UTR riboswitches in *G. theta* are affiliated with multi-exon genes, but only the *SSSQ* riboswitch is located near introns. *SSSQ* pre-mRNAs have two spliceoforms, with one or two introns splitting the riboswitch sequence (Figure 2c), suggesting that this riboswitch cannot operate before splicing occurs.

### Thiamine deprivation responses of prasinophytes and their novel riboswitch-containing genes

**Figure 3:**
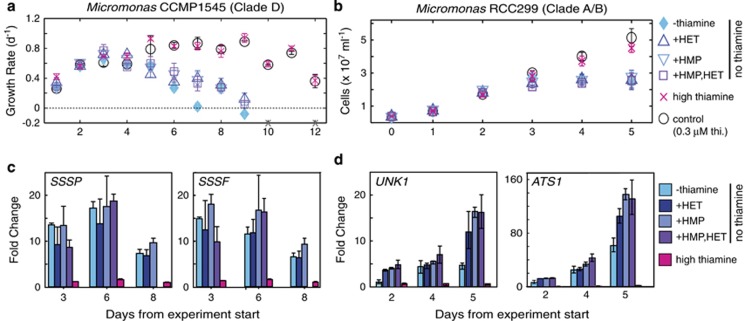
The novel riboswitch-containing genes are highly expressed under thiamine depletion. Growth of (**a**) *Micromonas pusilla* CCMP1545 and (**b**) *Micromonas* sp. RCC299 ceased in the absence of thiamine. (**c**) CCMP1545 and (**d**) RCC299 novel gene expression in all no-thiamine treatments increased significantly over positive controls while fold changes were minimal in high thiamine (1 μM, CCMP1545; 10 μM, RCC299). Note that due to the magnitude of gene expression change in thiamine-deplete treatments, qPCR bars/values are difficult to see for the high thiamine treatments in panel d (but are present; see Supplementary Tables S5 and S6a, b for values and additional time points). Error bars represent the mean and standard deviation for biological triplicates.

We performed thiamine-deprivation experiments to test B_1_ responsiveness of the novel algal riboswitch-containing genes in two *Micromonas* species. The species used have either *SSSP* and *SSSF* (CCMP1545) or *ATS1* and *UNK1* (RCC299). QPCR primer-probe sets were designed to these genes, *SSUA/THI5*-like genes and house-keeping genes (Supplementary Table S4). During preliminary follow-out experiments, CCMP1545 did not reach thiamine limitation in no-thiamine treatments (including those with precursor amendments) until the point where other medium constituents were limiting, complicating data interpretation. Therefore, CCMP1545 was transferred to maintain the initial cell concentration (daily, if needed), avoiding depletion of other constituents as well as induction of lag phase (which occurs if cells are transferred to too low concentration). For both *Micromonas* species, growth in positive controls (0.3 μM thiamine, the standard media concentration) was not significantly different from high thiamine treatments (1 μM, CCMP1545; 10 μM, RCC299, Figures 3a and b). In transfer experiments, both species ceased growing in treatments lacking thiamine (Figure 3a; Supplementary Figure S2a and b). Likewise, growth ceased in all no-thiamine treatments from the RCC299 follow-out experiment (Figure 3b). Maximum CCMP1545 and RCC299 cell yields in no-thiamine treatments were 1.1 × 10^8^±1.0 × 10^7^ and 2.4 × 10^7^±8.2 × 10^5^ cells ml^−1^, respectively.

Expression of the novel riboswitch-containing genes increased significantly in all no-thiamine treatments. In contrast, expression in positive controls and high thiamine remained low (Figures 3c and d). In all treatments without thiamine, RCC299 *ATS1* was significantly upregulated after 4 h and by >10-fold on day 2 (Figure 3d, Supplementary Table S5). At the final time point, *ATS1* expression in the no-thiamine treatment was ⩾80-fold higher than positive controls, and >100-fold higher in precursor treatments (which also lacked thiamine). A similar differential in response to precursor treatments versus no-thiamine alone was observed for *UNK1*, which had >10-fold higher expression in precursor treatments than positive controls at the final time point (Figure 3d). A third RCC299 riboswitch-containing gene (*SSUA/THI5*-like) had lower expression than either novel gene (Supplementary Table S5). In CCMP1545 (where *SSUA/THI5*-like does not have a riboswitch), this gene showed maximally twofold increase relative to positive controls (Supplementary Tables S6a and b). In contrast, CCMP1545 *SSSP* and *SSSF* expression was >10-fold higher than positive controls by day 2 in all treatments without thiamine, and remained high through day 6 (Figure 3c).

### *Relationships between TPP riboswitch-containing genes and B*_*1*_*biosynthesis enzymes in algae*

To better understand the thiamine biosynthetic capacity of algae harboring the novel riboswitch-containing genes, we investigated distributions of known ‘classical' thiamine pathway genes (Figure 1; Supplementary Table S3). Phytoplankton absent from prior algal TPP pathway studies (Croft *et al.,* 2006; Bertrand and Allen, 2012) were included by analyzing genomes from the open-ocean rhizarian *B. natans*, the cryptophyte *G. theta*, the chlorophyte *Volvox carteri* and the Class II prasinophyte *Ostreococcus* sp. RCC809, as well as transcriptomes from Class I (*P. parkeae* and *P. amylifera*) and Class II (*Micromonas* NEPCC29 and CCMP2099, representing *Micromonas* Clades C and E, respectively) prasinophytes.

**Figure 4:**
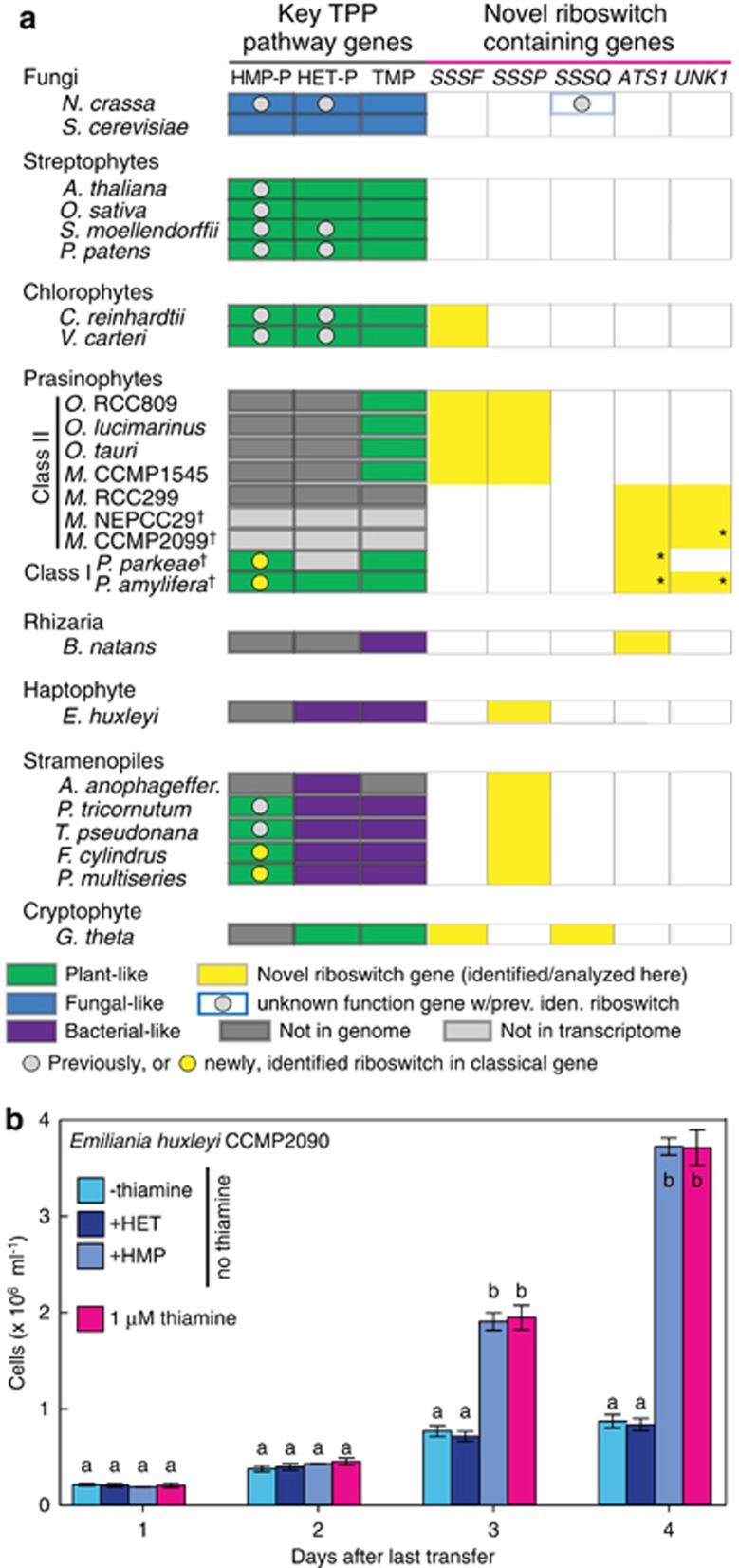
Classical vitamin B_1_ biosynthesis enzymes in phytoplankton and *Emiliania huxleyi* growth in thiamine manipulation experiments. (**a**) Presence or absence is indicated for genes encoding known enzymes (Figure 1; Supplementary Table S3) for HET-P (THI1/4, THIG) and HMP-P (THIC, THI5) synthesis as well as condensation to TMP (TH1 or THI6) that were plant-like (THI1/4, THIC and TH1; green), fungal (THI4, THI5 and THI6; blue), or bacterial-like (THIG, THIC and THIE/TH1; purple). Genomes were used for all gene (dark gray, absence) and riboswitch identifications except those from (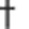) transcriptomes (where absence is inconclusive, light gray). Class II prasinophytes are also known as Mamiellophyceae (Marin and Melkonian, 2010). *Contigs ended before the UTR hence riboswitch presence is suspected, but unknown. Other symbols/colors are as indicated on figure. Riboswitches were also found in other phytoplankton genes (Supplementary Table S2). (**b**) *Emiliania huxleyi* growth in no-thiamine medium amended with HMP was not statistically different from positive controls, while growth ceased in thiamine-deplete medium amended with HET and negative controls. One-way ANOVAs were performed within each time point, letters represent groups with significant statistical differences (*post hoc* comparison by Holm–Sidak test: *P*<0.001).

Class II prasinophytes have multiple novel riboswitch-containing genes, but HMP-P (THIC) and HET-P (THI1/THI4) synthesis enzymes were absent from all species investigated (Figure 4a; Supplementary Table S3). *Ostreococcus* spp. and *Micromonas* Clade D (CCMP1545) have *SSSP* and *SSSF* genes. The latter is collocated with *TH1* encoding a plant-like bi-functional HMP-P kinase/TMP synthase and *THIM* (Supplementary Figure S4). In contrast, *Micromonas* species with ATS1 and UNK1 also lack THIM and TH1 homologs.

Among other green algae, complete plant-like thiamine biosynthesis pathways were present. *THIC* (with a riboswitch), *THI4*, and all other plant-like enzymes were detected in one or both Class I prasinophyte transcriptomes (Figure 4a; Supplementary Table S3). *ATS1* and *UNK1* were also present but in contigs lacking UTR sequence (preventing possible riboswitch identification). The chlorophytes *Chlamydomonas reinhardtii* and *V. carteri* have complete classical thiamine biosynthesis pathways and riboswitch-bearing *SSSF* genes (Figure 4a; Supplementary Table S3).

TPP biosynthesis pathways in lineages with plastids of red algal origin (for example, cryptophytes, stramenopiles and haptophytes) contained several enzymes different from green-lineage organisms and sometimes from each other (Figure 4a; Supplementary Table S3). *G. theta* had the most similarities to green lineage organisms. Most other red-lineage algae analyzed contained THIG, responsible for HET-P synthesis in bacteria (Figure 1) as well as a TH1 protein with highest identity to bacterial, not plant, homologs. They also lacked THIM (Supplementary Table S3). Diatoms were the only marine group with a complete classical pathway. Both *G. theta* and the haptophyte *E. huxleyi* have nearly complete pathways (albeit composed of different enzymes), lacking only a known HMP-P synthesis enzyme. Finally, each representative red-lineage taxon had at least one of the three riboswitch-containing putative sodium:solute symporter genes, with *SSSP* having the broadest distribution across the algae investigated (Figure 4a).

Homologs of bacterial thiamine transporter components THIB, THIP and THIT were not detected among the algae investigated. Proteins similar to THIQ, the ATPase of *Salmonella typhimurium's* thiamine uptake system (Webb *et al.,* 1998), were present except in *O. lucimarinus*, *O. tauri* and *E. huxleyi*. Clear homologs of *S. cerevisiae* thiamine/HMP transporters were not detected. Prasinophytes (except *O. lucimarinus*) and chlorophytes have proteins belonging to PF02133, but functional roles are uncertain due to low identity (<30%, best *E*-value 10^−16^) to *S. cerevisiae* high- (THI10) and low- (THI72) affinity thiamine transporters.

### Emiliania huxleyi growth in thiamine deprivation experiments

Exogenous B_1_ has been reported as a growth requirement for *E. huxleyi* (Bertrand and Allen, 2012; Read *et al.,* 2013). However, based on our analyses, we hypothesized *E. huxleyi* is not a thiamine auxotroph but rather an HMP auxotroph (Figure 4a; Supplementary Table 3). We performed experiments on *E. huxleyi* CCMP2090 and CCMP1516 grown in the absence of thiamine but with the precursors HET or HMP, as well as in positive and negative controls. While *E. huxleyi* growth ceased in negative controls and HET-amended no-thiamine treatments, it was significantly higher in positive controls and in no-thiamine medium amended with HMP (Figure 4b; Supplementary Figure S3). Furthermore, between the latter two conditions, growth did not differ significantly.

### Relatedness and distribution of proteins encoded by two novel riboswitch-containing genes

**Figure 5:**
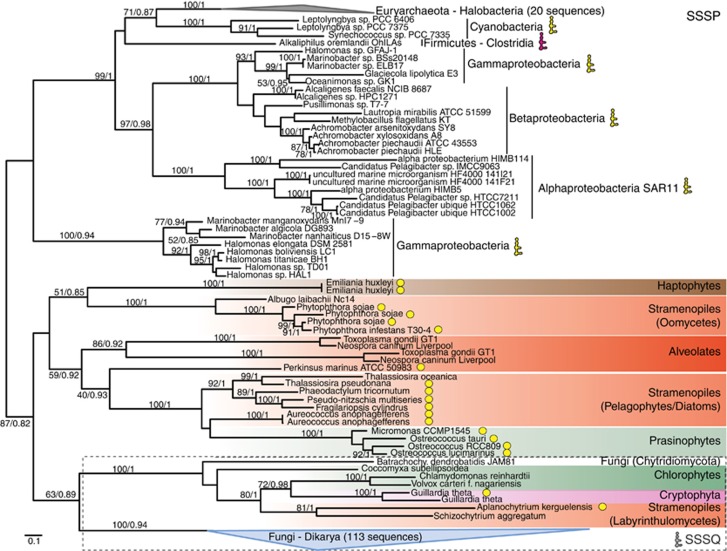
Maximum-likelihood analysis of SSSP and SSSQ proteins reveals relatedness and taxonomic distribution of these putative membrane transporters. Eukaryotic homologs encoded by genes containing riboswitches with structures resolved herein are indicated (yellow circles). Where symbols are absent we did not detect a riboswitch (note, fungal genes were not checked). Icons indicate riboswitch presence in (gray) the fungal homolog from *Neurospora crassa* (riboswitch-containing homolog NCU01977), (pink) a thiamine operon with a riboswitch several genes upstream from *SSSP* (Supplementary Figure S6b), and (yellow) clades where we identified riboswitches (always 5′ located) in representative taxa or predicted the secondary structure for one previously proposed in SAR11 (Meyer *et al.,* 2009; Worden *et al.,* 2009) (Supplementary Table S2; Supplementary Figure S6c). The overall relatedness of homologs was established in a larger scale reconstruction in which all sequences shown here belonged to a single bootstrap supported clade (Supplementary Figure S5). Node support ⩾50% (100 bootstrap replicates) and Bayesian posterior probabilities ⩾0.8 are shown.

The protein encoded by *SSSP* has the broadest distribution across the algae investigated and is the only novel riboswitch-containing gene identified in *E. huxleyi* (Figure 4a). BLAST results suggested it is related to SSSQ. Phylogenetic analyses confirms relatedness of these putative membrane transporters and demonstrate their presence in the three domains of life (Figure 5; Supplementary Figure S5). The SSSQ group contains 113 fungal homologs, including a putative transporter in *Neurospora crassa* with a TPP riboswitch (Li and Breaker, 2013), as well as chlorophyte, cryptophyte and labyrinthulomycete sequences (see Supplementary Figure S6a for the uncollapsed tree). Searches of all non-fungal *SSSQ* genes revealed affiliated riboswitches only in the marine species *G. theta* and *A. kerguelensis*. Most other marine algal and bacterial sequences could be recovered using *M. pusilla* CCMP1545 SSSP as a query in pairwise comparisons and formed several SSSP clades. Among *SSSP* genes, riboswitches were not detected in representative Euryarchaeota-Halobacteria clade species (Supplementary Figure S6a). *Alkaliphilus oremlandii* also lacks an *SSSP*-affiliated riboswitch, but we observed a TPP riboswitch at the start of the *THIE*, *THIM*, *THID* and *SSSP* operon (Supplementary Figure S6b). Riboswitches with robust secondary structure predictions were identified in representatives from other SSSP bacterial clades, including SAR11 isolate ‘*Candidatus* Pelagibacter ubique' (Supplementary Figure S6c), and all algal *SSSP* genes (Supplementary Table S2).

## Discussion

### Riboswitches are widespread across phytoplankton lineages

Vitamin B_1_ availability has been considered a major factor in shaping eukaryotic phytoplankton community composition and marine blooms (Tang *et al.,* 2010; Bertrand and Allen, 2012). Recent reports of low B_1_ levels in surface waters further substantiated this concept (Sanudo-Wilhelmy *et al.*, 2012; Carini *et al.,* 2014). Thus, efficient regulation of B_1_ biosynthesis pathways (and related transport mechanisms) by TPP riboswitches would seem advantageous in marine environments. We have discovered riboswitches in multiple eukaryotic lineages not known to undergo this form of gene regulation, specifically cryptophyte, haptophyte, rhizarian and alveolate phytoplankton (Figure 2a). Riboswitches were also identified in Class I prasinophytes and several stramenopiles. Our results expand prior observations from green algae, diatoms, plants and fungi (Cheah *et al.,* 2007; Croft *et al.*, 2007; Worden *et al.,* 2009; Wachter, 2010). We conclude that riboswitches have a broad taxonomic distribution in marine phytoplankton.

Secondary structures of the predicted riboswitches have hallmarks of TPP riboswitches, suggesting that they are likely functional (Figure 2b; Supplementary Figure S1). Most share extensive conservation with plant riboswitches although *G. theta* (Figure 2b) and *M. pusilla* CCMP1545 have divergent P4 U-A pairing (Supplementary Figure 1b) also present in confirmed fungal riboswitches (Cheah *et al.,* 2007). Collectively, algal P3 stem lengths encompass a broader range than the 17–60 bases observed for plant riboswitches (Wachter *et al.,* 2007). This presumably does not affect function since overall P3 length does not appear to inhibit riboswitch activity in model taxa (Wachter, 2010).

Our results show that variability in riboswitch positioning (along genes) is relatively common, even among closely related taxa (Figure 2c; Supplementary Table S2). Additionally, many are located in 5′ UTRs of single exon genes with no apparent alternative spliceoforms. This is unlike riboswitches in plants and fungi, which are present in multi-exon genes and induce alternative splicing or intron retention (Cheah *et al.,* 2007; Wachter *et al.,* 2007). As such, several riboswitches identified here may operate in an as yet undocumented manner, or possibly akin to some bacterial riboswitches which interfere with translation by blocking ribosome binding (Breaker, 2011).

In plants and fungi, most TPP riboswitches are associated with the HMP-P biosynthesis genes *THIC* and *THI5*, respectively, as well as *THI4*, encoding the HET-P biosynthesis enzyme in fungi (Cheah *et al.,* 2007; Wachter *et al.,* 2007) (Figure 1). In non-vascular plants and chlorophyte algae, *THIC* and *THI1/THI4* have riboswitches (Wachter, 2010). *THIC* riboswitches are also known in two diatoms (Croft *et al.*, 2007). Here, we identified *THIC* riboswitches in prasinophytes (the first *THIC* genes reported in this group), and in additional diatom species (Figure 4a), indicating riboswitch control of HMP-P synthesis genes is taxonomically widespread. We also identified a riboswitch within a eukaryotic *THIM* gene, an association otherwise only shown in bacteria (Winkler and Breaker, 2005).

### Identification of ‘new' thiamine-responsive genes

Strikingly, the majority of riboswitches discovered here were in genes with poorly characterized functions that have not been experimentally connected to thiamine pathways. Distributions of some novel riboswitch-containing genes transcend major eukaryotic divisions (Figure 4a). Of the taxa analyzed, the Mamiellophyceae have the most, but in mutually exclusive sets. Moreover, the Mamiellophyceae appear to have undergone extensive reduction in classical pathway components. Thus, while some of the novel riboswitch-containing genes are present in prototrophs investigated here, they are numerically more prevalent in taxa missing two or more enzymes involved in classical pathways (Figure 4a).

We tested thiamine growth responses and novel riboswitch-containing gene expression using two *Micromonas* species. These species share at most 90% of their protein encoding genes based on genome analyses (Worden *et al.,* 2009). That these picoeukaryotes (⩽2 μm diameter) are missing classical enzymes required for biosynthesis of both precursors (Figure 4a) is surprising because they grow in oligotrophic settings (Treusch *et al.,* 2012) where *de novo* synthesis would seem advantageous. Moreover, even though *M. pusilla* CCMP1545 and related picoeukaryotes, *Ostreococcus* spp., lack known enzymes for precursor moiety synthesis, they possess TMP synthase (TH1, Figure 1). Based on the observed pathway composition (and gaps), we included thiamine deprivation treatments with precursor supplementation. Both *Micromonas* species ultimately ceased growing in treatments without thiamine (Figures 3a and b). Combined with pathway analyses these results suggest that RCC299 and CCMP1545 are thiamine auxotrophs, unless one or both precursor compounds used here were not the required form. However, CCMP1545 produced an order of magnitude more cells than RCC299, implying differences in overall thiamine requirements that could influence population dynamics and competition processes in the ocean. The gene expression results from experiments on both species unequivocally demonstrated the novel riboswitch-containing genes are highly responsive to thiamine deprivation (Figures 3c and d).

### Thiamine auxotrophy or precursor auxotrophy?

Several recent studies conclude that B_1_ availability controls phytoplankton growth, harmful algal blooms, and species succession, based on either analyses of known thiamine biosynthesis enzymes or thiamine deprivation experiments (Croft *et al.,* 2006; Tang *et al.,* 2010; Bertrand and Allen, 2012; Read *et al.,* 2013). We considered whether retention of most, but not all, known enzymes might allow for B_1_ biosynthesis using alternative steps (potentially involving the novel riboswitch-containing genes) since most taxa investigated have TMP synthase and TPK1, the kinase responsible for TMP phosphorylation (Figure 1; Supplementary Table S3). Rather than being thiamine auxotrophs, they could be auxotrophic for a precursor moiety (or both). Depending on gene presence/absence patterns, exogenous precursor supplies would be used or, possibly, as yet unidentified compounds. Therefore, we hypothesized *E. huxleyi* can grow using HMP alone. The hypothesis was proven by our experiments which demonstrate HMP obviates *E. huxleyi*'s need for exogenous thiamine. Moreover, there was no statistical difference in growth in HMP amended medium versus thiamine amended medium. These results suggest that B_1_ scarcity does not control blooms of this primary producer if HMP is available.

Mechanisms for uptake and growth on HMP may be common. Although no replication was used, early experiments on the haptophyte *Prymnesium parvum* and chrysophyte *Monochrysis lutheri* indicated that cultures deprived of thiamine grew better when amended with 4-amino-5-aminomethyl-2-methylpyrimidine (AmMP, an HMP analog) than without (Droop, 1958). Additionally, thiamine auxotrophic mutants of the model prototrophs *Salmonella typhimurium* and *Saccharomyces cerevisiae* can grow in HMP amended media (Newell and Tucker, 1966; Nosaka, 2006). Thus, combined with our results, capacity for growth using just the pyrimidine portion of thiamine may be common. Indeed, members of the marine heterotrophic bacterial group SAR11 grow on HMP (Carini *et al.,* 2014). Above 100 pM, HMP supports significant SAR11 growth, while pM and nM thiamine and AmMP concentrations do not. In marine surface waters B_1_ concentrations are often below detection (<0.8 pM), in part because it is sensitive to photodegradation (Sanudo-Wilhelmy *et al.*, 2012). However, pyrimidines are chemically more stable than B_1_ (Carlucci *et al.,* 1969) and can exceed thiamine concentrations in the oligotrophic ocean (Carini *et al.,* 2014), enhancing their general availability. Thus, although largely neglected in recent oceanographic studies, pathway intermediates such as HMP appear to be utilized by both heterotrophic bacteria and eukaryotic phytoplankton.

The transport mechanism for HMP acquisition remains unclear in *E. huxleyi*, other marine algae and SAR11. In *S. cerevisiae*, one or more of three different thiamine transporters is thought to be responsible for HMP acquisition (Nosaka, 2006). We found only one *E. huxleyi* protein related to a known *S. cerevisiae* thiamine/HMP transporter. However, similarity was low (25%) between the *E. huxleyi* protein and *S. cerevisiae* THI73, a low-affinity thiamine transporter thought to have other primary roles (Nosaka, 2006). Interestingly, like *E. huxleyi*, the SAR11 thiamine biosynthesis pathway lacks only an enzyme for HMP synthesis (Carini *et al.,* 2014) and has *SSSP* with an associated riboswitch (Worden *et al.,* 2009). *SSSP* has been termed *THIV* in *Methylobacillus flagellatus*, where its function is unknown but a TPP riboswitch is also present (Rodionov *et al.,* 2002). Similar to observations by Carini and colleagues, we noted *SSSP* is collocated with *THIG* in several Marinobacters and present in an *A. oremlandii* thiamine biosynthesis operon (Supplementary Figure S6b). The presence of *SSSP* as well as *SSSF* (Supplementary Figure S4) in genomic neighborhoods containing TPP biosynthesis pathway genes is strongly suggestive of thiamine-related roles, potentially in uptake of precursor compounds.

The distribution of SSSP/SSSQ across archaea, bacteria and diverse eukaryotic lineages, as well as the frequency with which TPP riboswitches are found (Figure 5), underscore a long-standing connection between the riboswitch and this putative membrane transporter. We postulate SSSP/SSSQ transporters were part of an early thiamine biosynthesis pathway and have deeper evolutionary roots than some classical pathway components with narrower distributions in extant eukaryotes. We hypothesize that SSSP has thiamine or HMP transport functions while Carini *et al.* (2014) propose it transports HMP or AmMP. Once directed, manipulatable genetic systems are available for SAR11, or algal genera like *Emiliania* and *Micromonas*, it should be possible to further assess functional roles.

## Conclusions

Breaker (2011) asked ‘Where are the eukaryotic riboswitches?' because their distribution in eukaryotes appeared to be very limited. Here, we have shown riboswitches are present in representatives from all major eukaryotic marine phytoplankton lineages. The structures identified have high conservation with verified plant TPP riboswitches, but are primarily associated with novel unknown function genes not previously linked to thiamine metabolism. These genes are significantly upregulated under thiamine deprivation. However, many of the eukaryotic riboswitches described here are linked to single-exon genes. Therefore, bacterial-like riboswitch activities, or possibly other unknown modalities, appear widespread in unicellular eukaryotes whereas regulation by alternative splicing likely emerged later in eukaryotic evolution. These results provide exploratory targets for biotechnological innovations (Breaker, 2011; Ramundo *et al.,* 2013), unknown aspects of B_1_ pathways and variations in RNA-based controls of eukaryotic gene regulation.

A longstanding question in oceanography is how vitamins influence primary production and phytoplankton succession (Sanudo-Wilhelmy *et al.*, 2012). Our studies on ecologically relevant phytoplankton highlight how examination of diverse taxa may reveal new aspects of pathways established in model taxa. We demonstrate that the major primary producer *E. huxleyi* grows on the pathway intermediate HMP, although formerly characterized as having an incomplete pathway and requiring thiamine. Our results highlight the need to quantify pathway intermediates in seawater alongside end-products like vitamin B_1_. Thus, this study underscores the importance of reformulating paradigms on vitamin regulation of phytoplankton growth—and microbial communities in general—to incorporate the role of exogenous pathway intermediates.
