# Supplementary material for: Alternatives to vitamin B1 uptake revealed with discovery of riboswitches in multiple marine eukaryotic lineages
Source: ISME J. 2014 Aug 29;8(12):2517–29. doi: 10.1038/ismej.2014.146 (PMC4260697; doi:10.1038/ismej.2014.146)
Supplement: Supplementary Information [file ismej2014146x1.pdf]

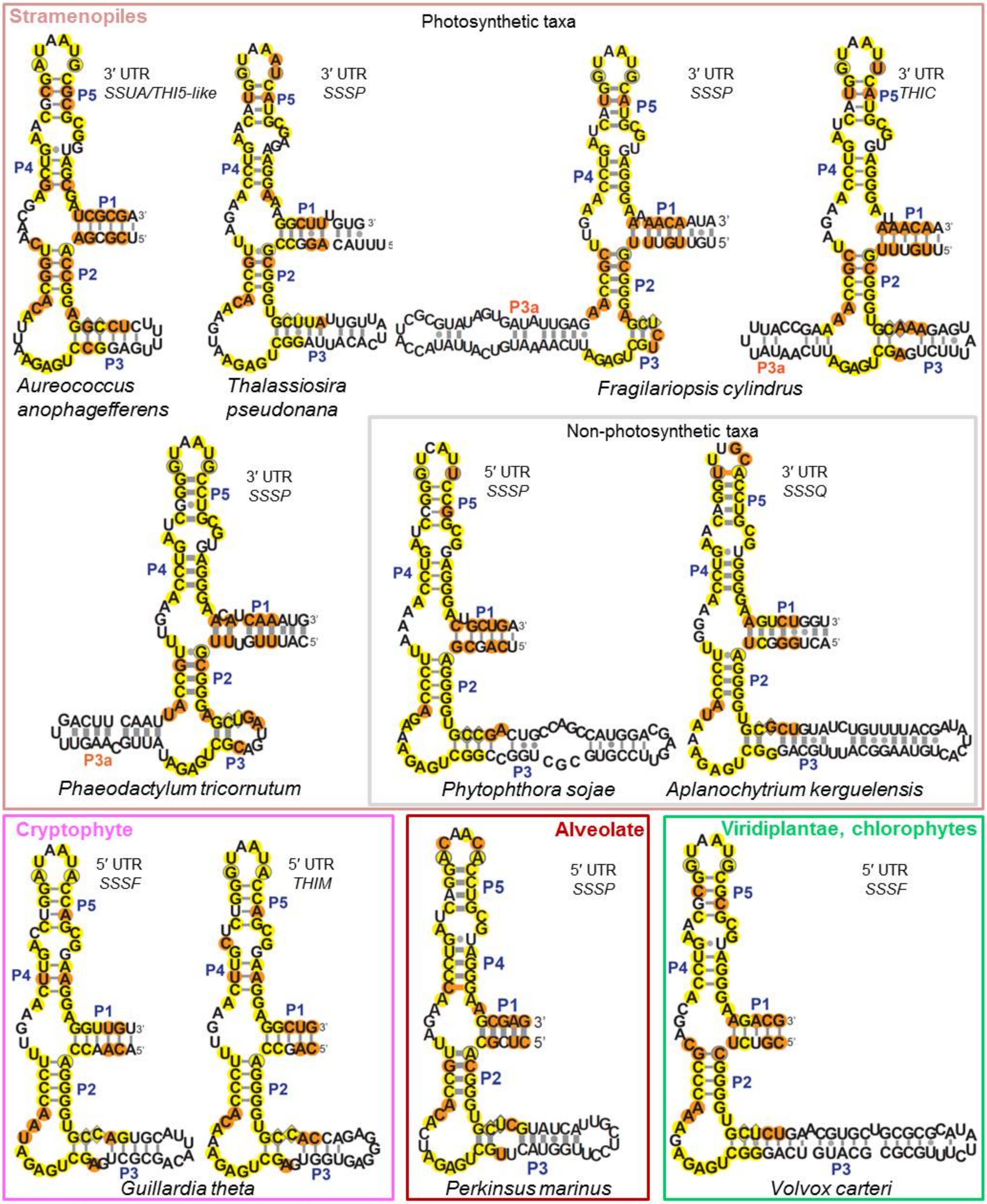
Stramenopiles
Photosynthetic taxa
3′ UTR SSUA/THI5-like
Aureococcus anophagefferens
3′ UTR SSSP
Thalassiosira pseudonana
3′ UTR SSSP
Fragilariopsis cylindrus
3′ UTR THIC
3′ UTR SSSP
Phaeodactylum tricornutum
Non-photosynthetic taxa
5′ UTR SSSP
Phytophthora sojae
3′ UTR SSSQ
Aplanochytrium kerguelensis
Cryptophyte
5′ UTR SSSF
5′ UTR THIM
Guillardia theta
Alveolate
5′ UTR SSSP
Perkinsus marinus
Viridiplantae, chlorophytes
5′ UTR SSSF
Volvox carteri
P1
P2
P3
P3a
P4
P5

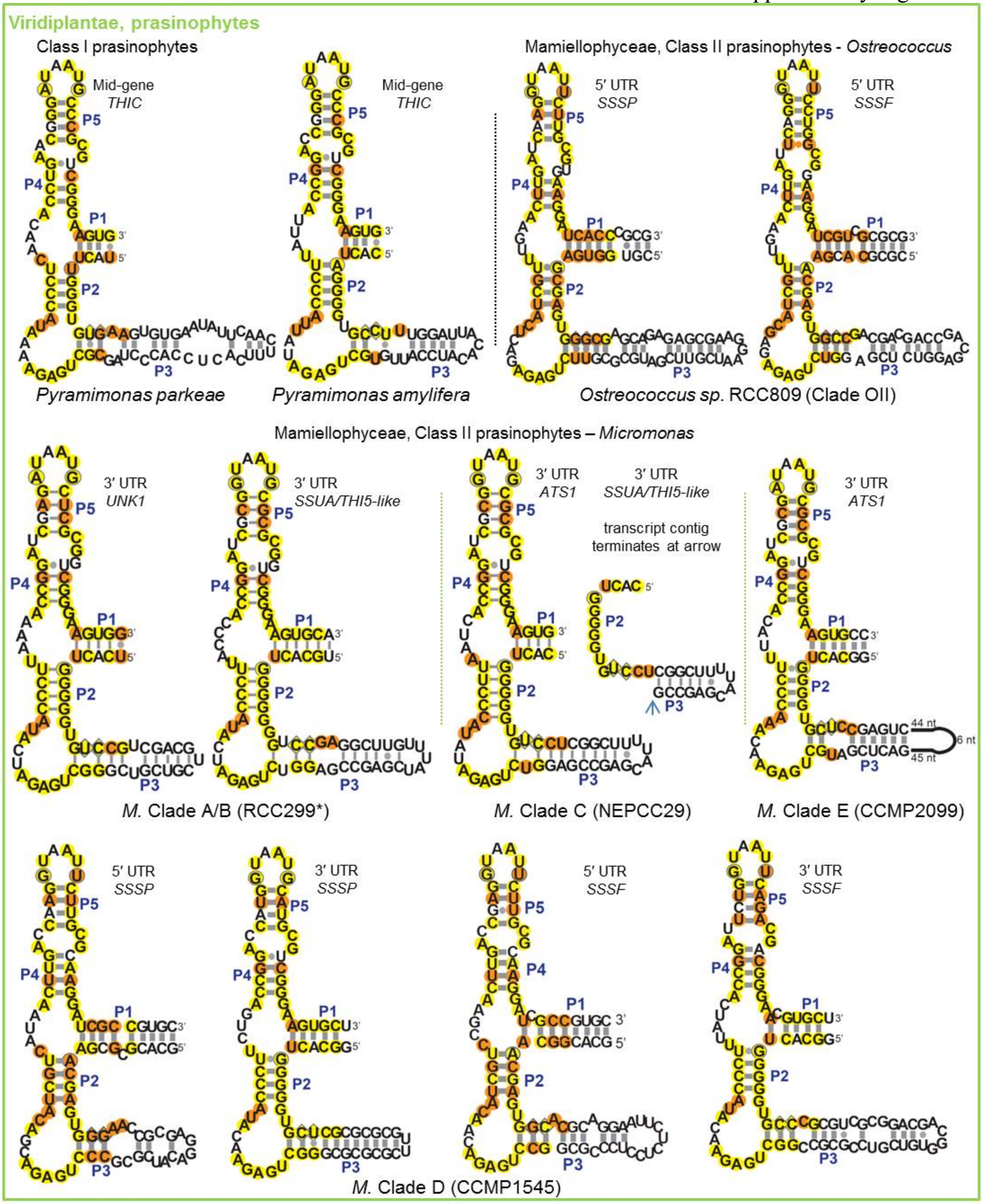
Viridiplantae, prasinophytes
Class I prasinophytes
Mid-gene THIC
Mid-gene THIC
Pyramimonas parkeae
Pyramimonas amylifera
Mamiellophyceae, Class II prasinophytes - Ostreococcus
5′ UTR SSSP
5′ UTR SSSF
Ostreococcus sp. RCC809 (Clade OII)
Mamiellophyceae, Class II prasinophytes – Micromonas
3′ UTR UNK1
3′ UTR SSUA/THI5-like
M. Clade A/B (RCC299*)
3′ UTR ATS1
3′ UTR SSUA/THI5-like
transcript contig terminates at arrow
M. Clade C (NEPCC29)
3′ UTR ATS1
44 nt
6 nt
45 nt
M. Clade E (CCMP2099)
5′ UTR SSSP
3′ UTR SSSP
5′ UTR SSSF
3′ UTR SSSF
M. Clade D (CCMP1545)
P1
P2
P3
P4
P5

Supplementary Figure S1. Newly predicted riboswitch structures not shown in Figure 2b. Conserved plant nucleobases (background circles) indicate those that are identical in the newly identified riboswitches (yellow) or diverged (orange) from plant. Plant positions that are conserved, but can be A or G (outlined circles), or C or U (outlined diamonds) in (Wachter *et al.* 2007), are also indicated. Apart from the 5′ (*SSSP*) and 3′ (*SSUA/THI5*-like) UTR riboswitches found in *A. anophagefferens* riboswitch locations within genes appeared to be consistent within each species (Supplementary Table S2). Riboswitches from three non-photosynthetic species are shown, the alveolate *Perkinsus marinus*, and the stramenopiles *Phytophthora sojae* and *Aplanochytrium kerguelensis*. These non-photosynthetic taxa are thought to have photosynthetic ancestors. Note that *P. sojae* has 3 highly similar copies of SSSQ (Protein IDs EGZ18412, EGZ18392, and EGZ29809), each with a riboswitch, but only one is shown here. Details are in Supplementary Table S2.

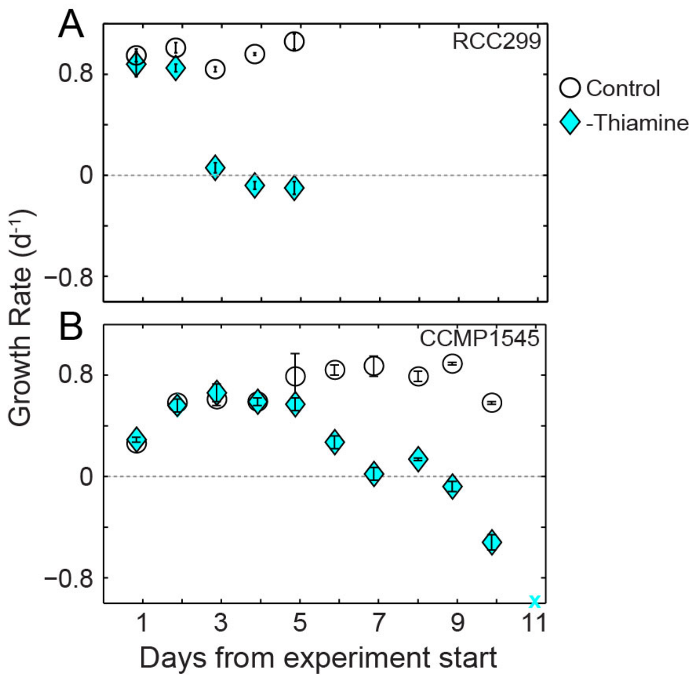

Supplementary Figure S2. Responses to thiamine deprivation in transfer experiments for **(a)** *Micromonas* RCC299 (Clade A/B) and **(b)** *Micromonas* CCMP1545 (Clade D). Cells were acclimated to thiamine replete conditions for ≥10 generations, replete medium was removed (cells were centrifuged and the supernatant discarded), cells were resuspended in the various treatment conditions and then transferred daily in no (blue diamonds) or 0.3 µM thiamine (open circles, control concentration) media. Error bars represent the SD for biological triplicates. A similar cessation and renewal of CCMP1545 growth at day 8 in no thiamine treatments was confirmed in another experiment performed in biological quadruplicate (qPCR results shown in Supplementary Table S6b). X denotes an off scale 'negative' CCMP1545 growth rate.

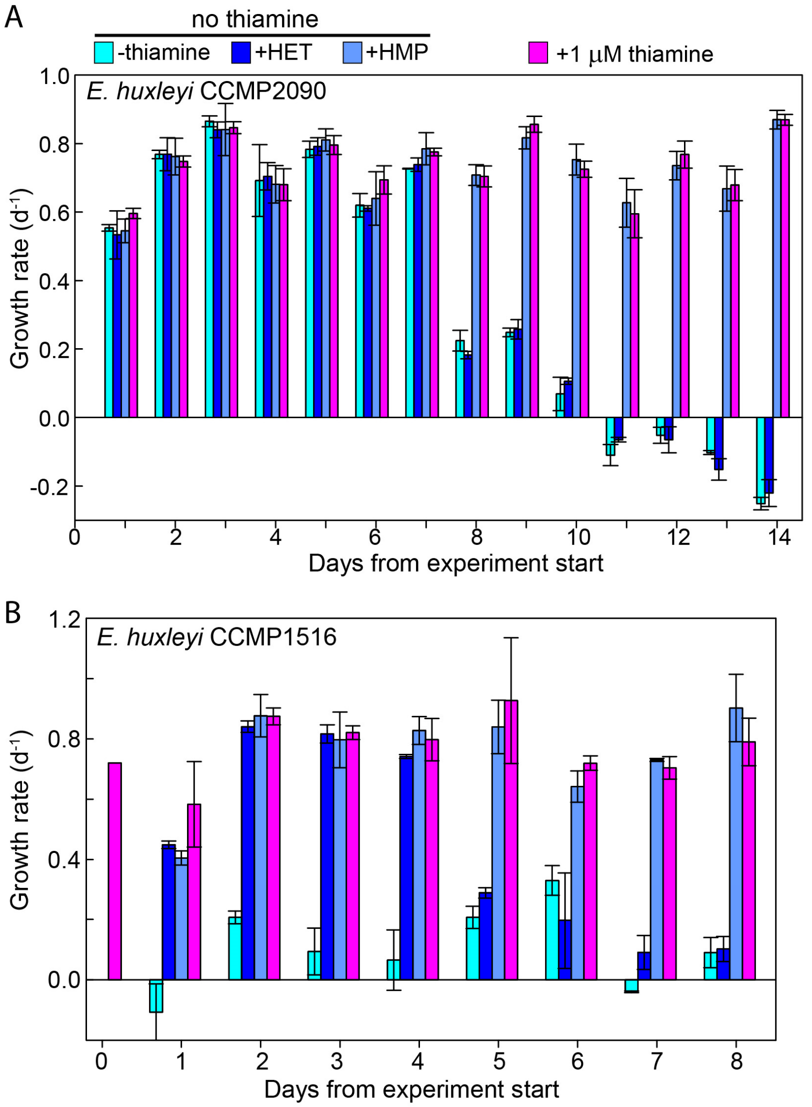

Supplementary Figure S3. Growth of two *E. huxleyi* strains is equivalent in medium amended with thiamine or HMP. **(a)** *E. huxleyi* CCMP2090 and **(b)** *E. huxleyi* CCMP1516 could not grow in the absence of thiamine unless HMP was added. Experiments were performed in biological triplicate and cells were transferred daily to the same concentration unless growth ceased, in which case transferring was terminated. There was no statistical difference for either strain between growth with i) thiamine or ii) HMP in the absence of thiamine. Growth rates decreased dramatically in treatments lacking thiamine or HMP. Note that DAPI results showed that CCMP1516 was not axenic (nor was it upon acquisition) and bacteria could be observed throughout the CCMP1516 experiment at <1 bacterium per eukaryotic cell, continued minor levels of growth throughout the experiment may be related to presence of bacteria.

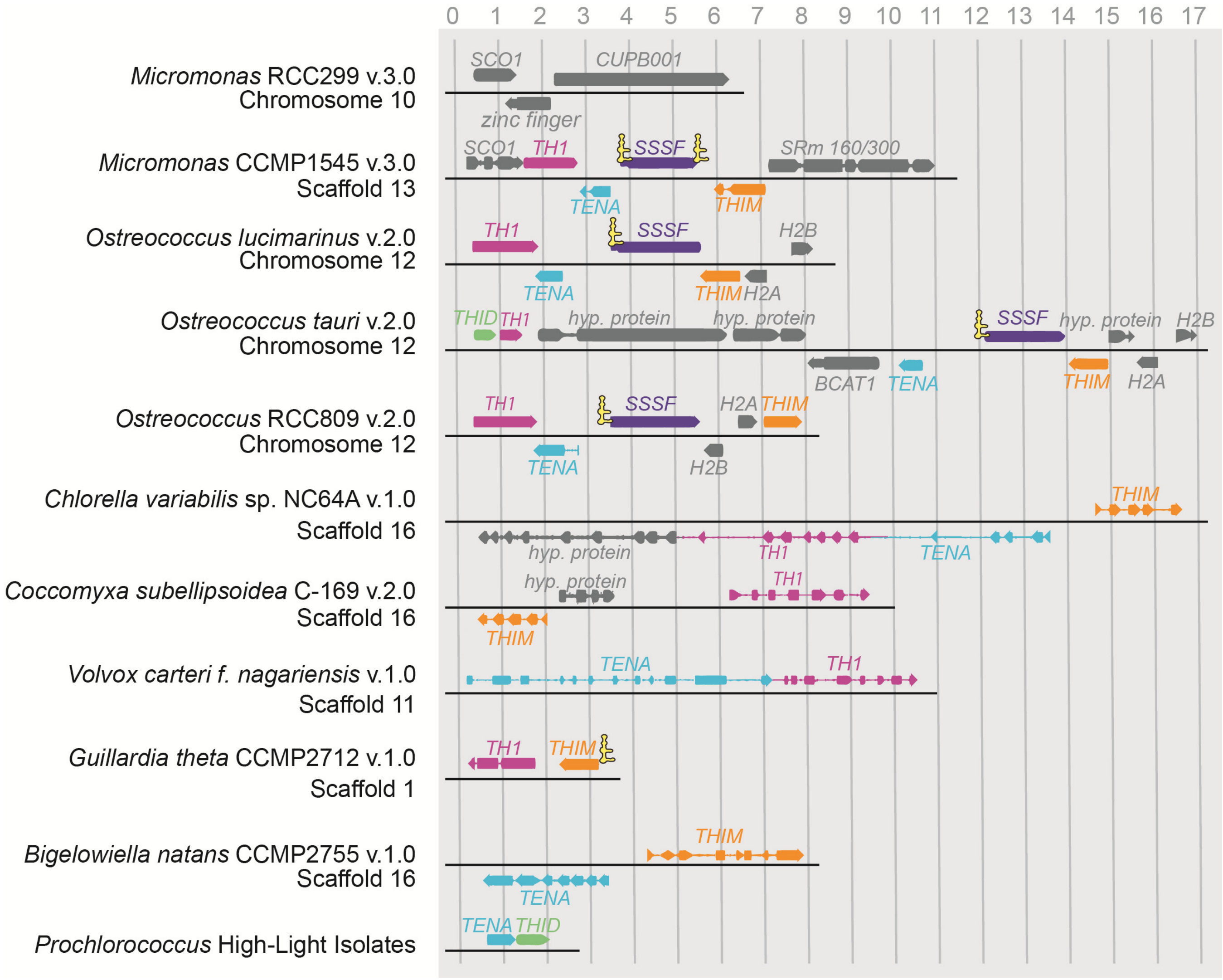

Supplementary Figure S4. Co-located TPP biosynthesis and *SSSF* genes in phytoplankton. Genes shown encode: TH1, HMP-P kinase and precursor condensation (pink); THIM, HET kinase (orange); THID, HMP-P kinase (green); and TENA, thiaminase (blue), SSSF (purple), and others not known to be involved in TPP biosynthesis (grey). Coding direction (relative to the plus strand) is indicated by arrowhead, exons by thicker lines, and introns by thinner lines. Putative riboswitches (yellow icons) are shown at approximate gene positions. *V. carteri* and *G. theta* both have *SSSF* on different scaffolds than the classical genes shown. Vertical lines show 1 kb increments from an arbitrary start point.

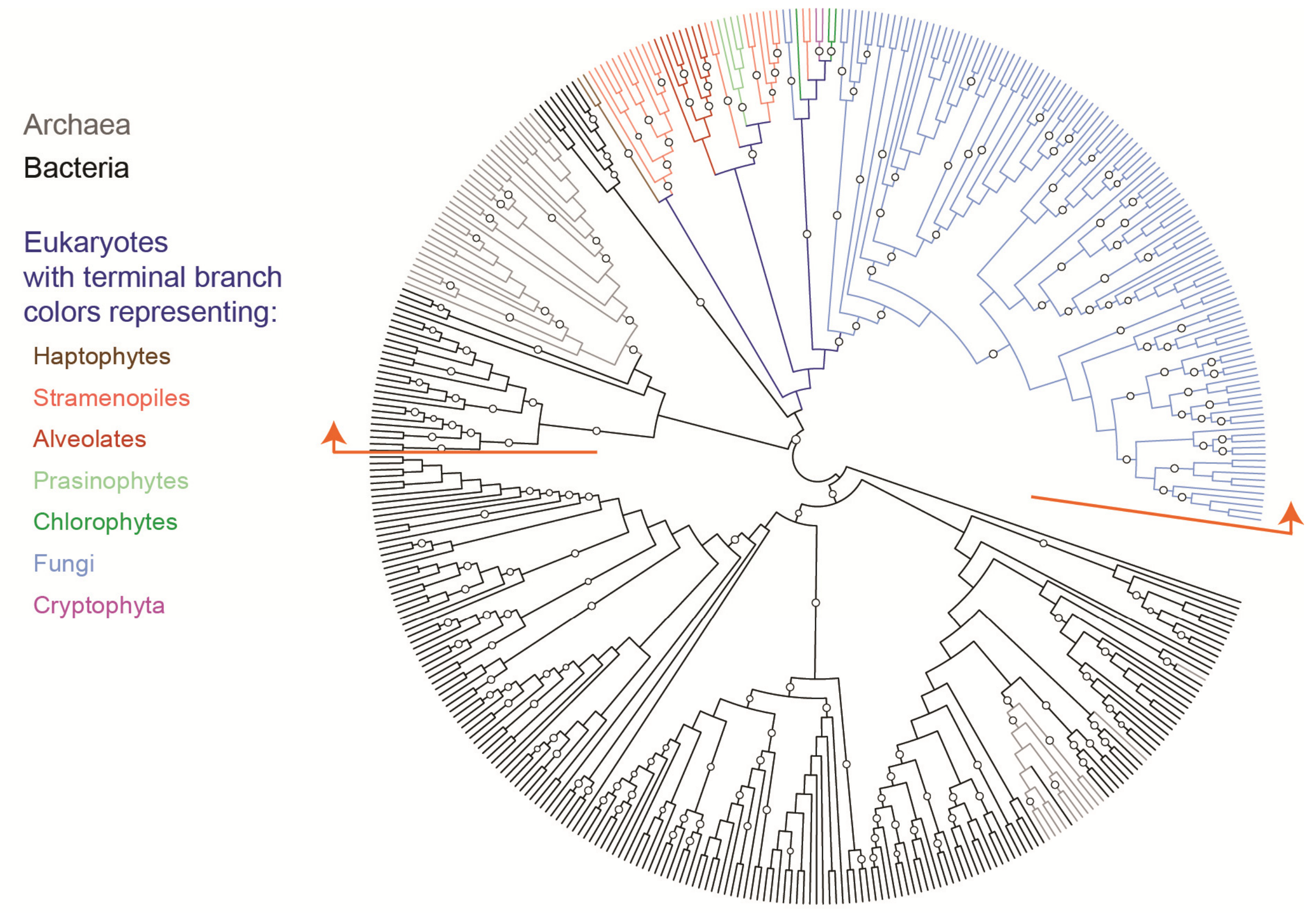

Supplementary Figure S5. Maximum likelihood analysis of sequences recovered in iterative BLASTP searches involving SSSP and SSSQ, which recruited some of the same hits. The algal sequences belonged to a large bootstrap supported clade (sequences demarked by orange arrows) that contained related bacterial and archaeal sodium:solute symporters and was further analyzed in Figure 5. 412 positions were used from an alignment of 413 protein sequences. Node support is based on 100 bootstrap replicates and indicated (open circles) where ≥70%.

Supplementary Figure S6a, Part I

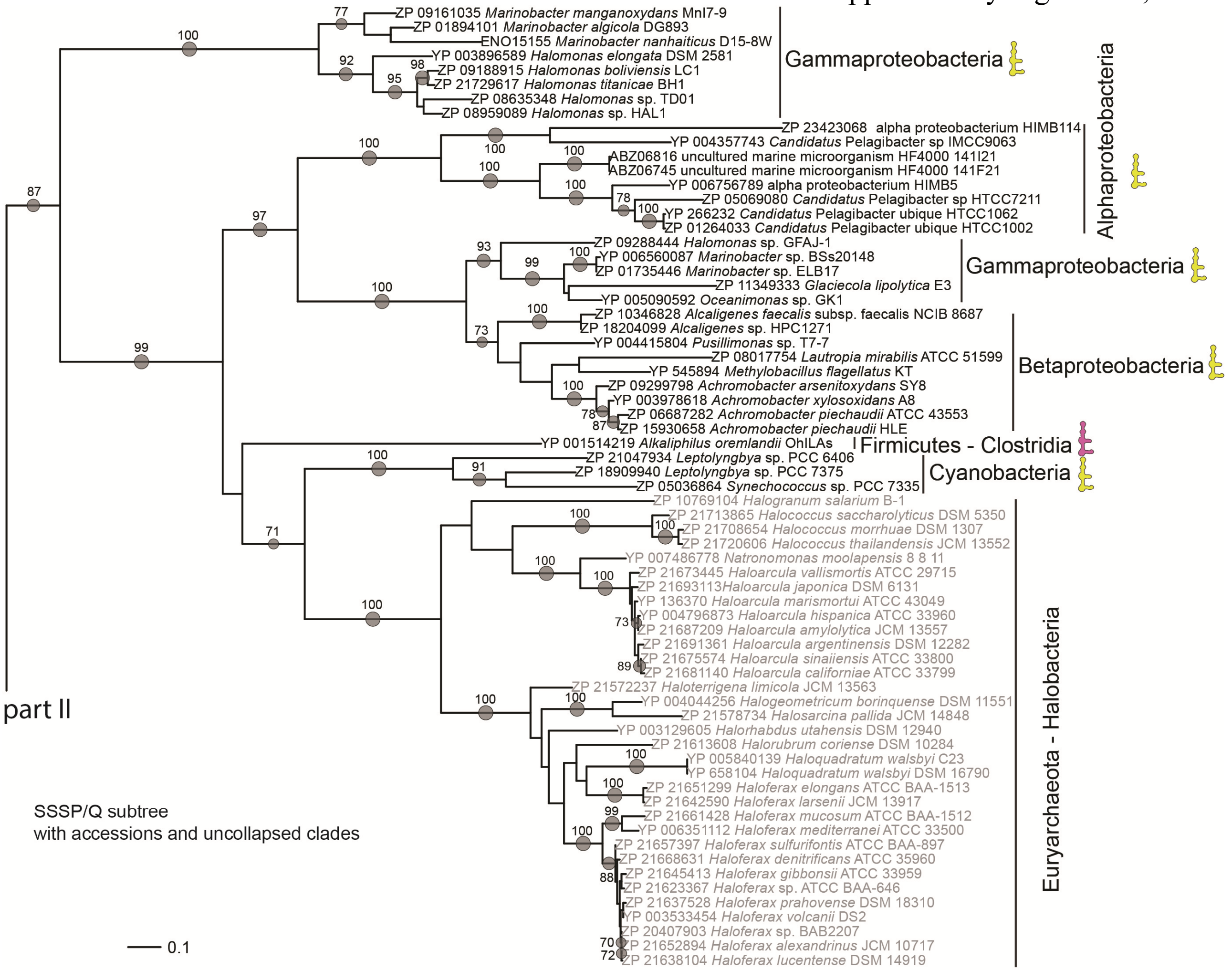

Supplementary Figure S6a continued, Part II

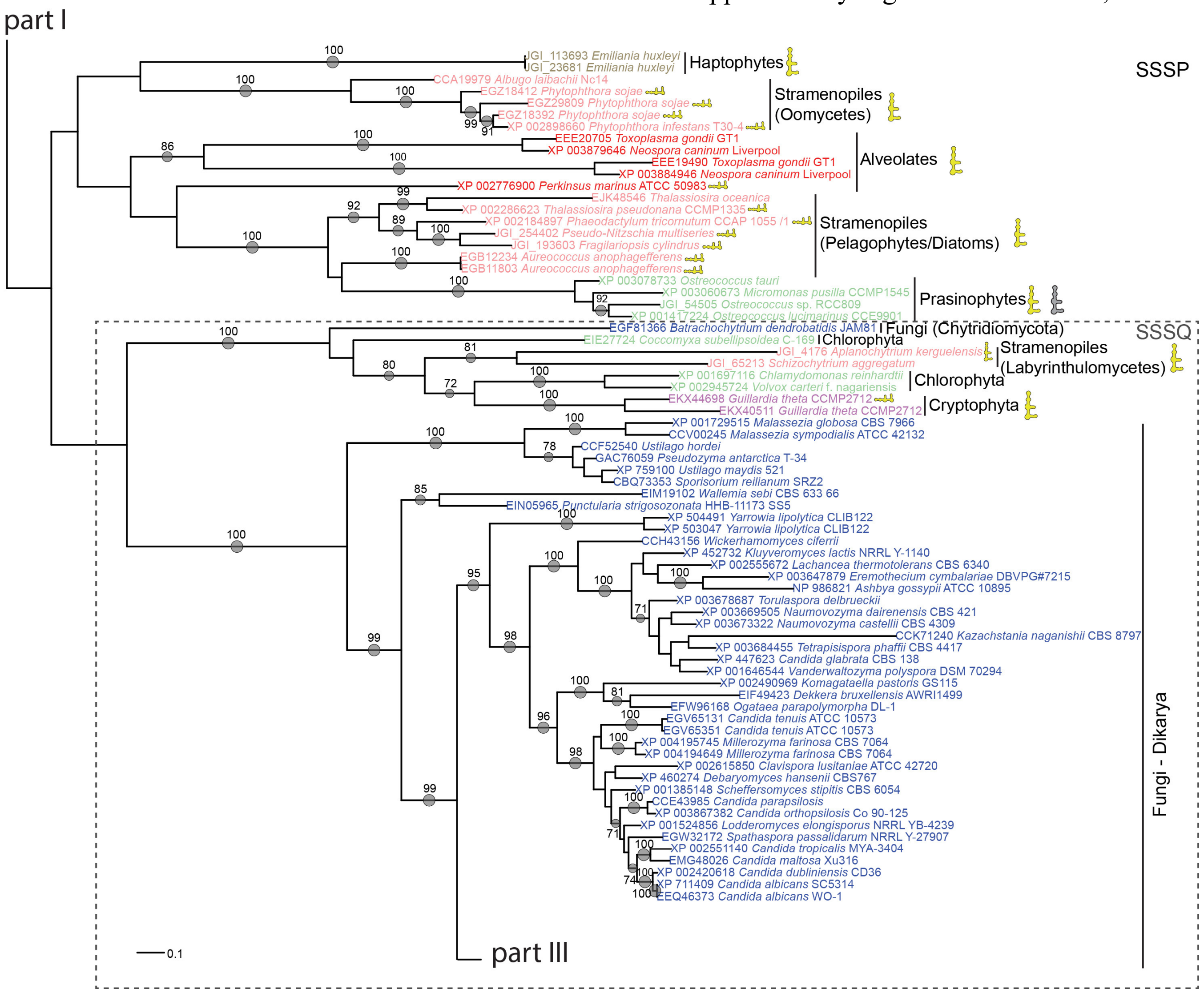

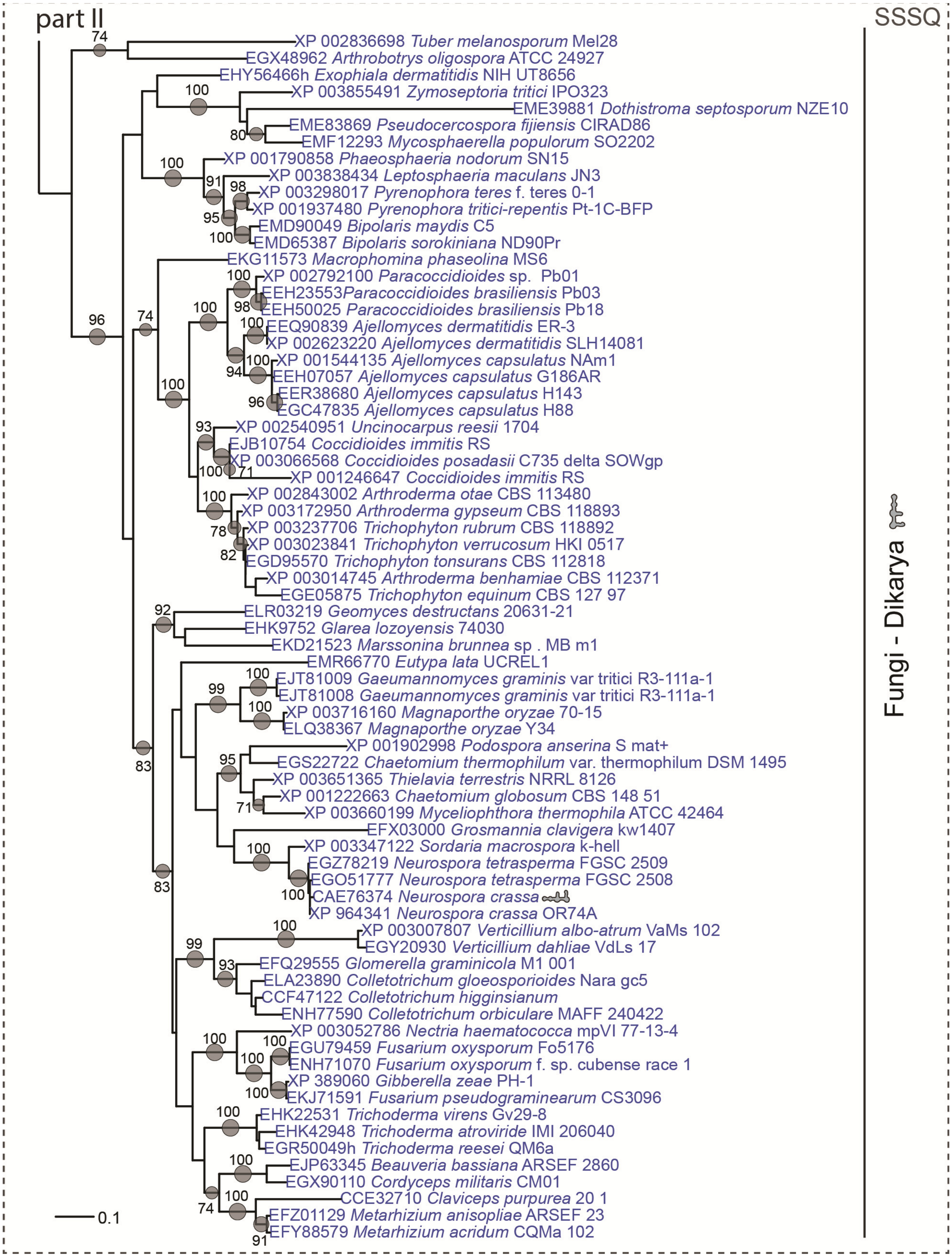
part II
SSSQ
XP 002836698 Tuber melanosporum Mel28
EGX48962 Arthrobotrys oligospora ATCC 24927
EHY56466h Exophiala dermatitidis NIH UT8656
XP 003855491 Zymoseptoria tritici IPO323
EME39881 Dothistroma septosporum NZE10
EME83869 Pseudocercospora fijiensis CIRAD86
EMF12293 Mycosphaerella populorum SO2202
XP 001790858 Phaeosphaeria nodorum SN15
XP 003838434 Leptosphaeria maculans JN3
XP 003298017 Pyrenophora teres f. teres 0-1
XP 001937480 Pyrenophora tritici-repentis Pt-1C-BFP
EMD90049 Bipolaris maydis C5
EMD65387 Bipolaris sorokiniana ND90Pr
EKG11573 Macrophomina phaseolina MS6
XP 002792100 Paracoccidioides sp. Pb01
EEH23553Paracoccidioides brasiliensis Pb03
EEH50025 Paracoccidioides brasiliensis Pb18
EEQ90839 Ajellomyces dermatitidis ER-3
XP 002623220 Ajellomyces dermatitidis SLH14081
XP 001544135 Ajellomyces capsulatus NAm1
EEH07057 Ajellomyces capsulatus G186AR
EER38680 Ajellomyces capsulatus H143
EGC47835 Ajellomyces capsulatus H88
XP 002540951 Uncinocarpus reesii 1704
EJB10754 Coccidioides immitis RS
XP 003066568 Coccidioides posadasii C735 delta SOWgp
XP 001246647 Coccidioides immitis RS
XP 002843002 Arthroderma otae CBS 113480
XP 003172950 Arthroderma gypseum CBS 118893
XP 003237706 Trichophyton rubrum CBS 118892
XP 003023841 Trichophyton verrucosum HKI 0517
EGD95570 Trichophyton tonsurans CBS 112818
XP 003014745 Arthroderma benhamiae CBS 112371
EGE05875 Trichophyton equinum CBS 127 97
ELR03219 Geomyces destructans 20631-21
EHK9752 Glarea lozoyensis 74030
EKD21523 Marssonina brunnea sp . MB m1
EMR66770 Eutypa lata UCREL1
EJT81009 Gaeumannomyces graminis var tritici R3-111a-1
EJT81008 Gaeumannomyces graminis var tritici R3-111a-1
XP 003716160 Magnaporthe oryzae 70-15
ELQ38367 Magnaporthe oryzae Y34
XP 001902998 Podospora anserina S mat+
EGS22722 Chaetomium thermophilum var. thermophilum DSM 1495
XP 003651365 Thielavia terrestris NRRL 8126
XP 001222663 Chaetomium globosum CBS 148 51
XP 003660199 Myceliophthora thermophila ATCC 42464
EFX03000 Grosmannia clavigera kw1407
XP 003347122 Sordaria macrospora k-hell
EGZ78219 Neurospora tetrasperma FGSC 2509
EGO51777 Neurospora tetrasperma FGSC 2508
CAE76374 Neurospora crassa
XP 964341 Neurospora crassa OR74A
XP 003007807 Verticillium albo-atrum VaMs 102
EGY20930 Verticillium dahliae VdLs 17
EFQ29555 Glomerella graminicola M1 001
ELA23890 Colletotrichum gloeosporioides Nara gc5
CCF47122 Colletotrichum higginsianum
ENH77590 Colletotrichum orbiculare MAFF 240422
XP 003052786 Nectria haematococca mpVI 77-13-4
EGU79459 Fusarium oxysporum Fo5176
ENH71070 Fusarium oxysporum f. sp. cubense race 1
XP 389060 Gibberella zeae PH-1
EKJ71591 Fusarium pseudograminearum CS3096
EHK22531 Trichoderma virens Gv29-8
EHK42948 Trichoderma atroviride IMI 206040
EGR50049h Trichoderma reesei QM6a
EJP63345 Beauveria bassiana ARSEF 2860
EGX90110 Cordyceps militaris CM01
CCE32710 Claviceps purpurea 20 1
EFZ01129 Metarhizium anisopliae ARSEF 23
EFY88579 Metarhizium acridum CQMa 102
Fungi - Dikarya
0.1

b

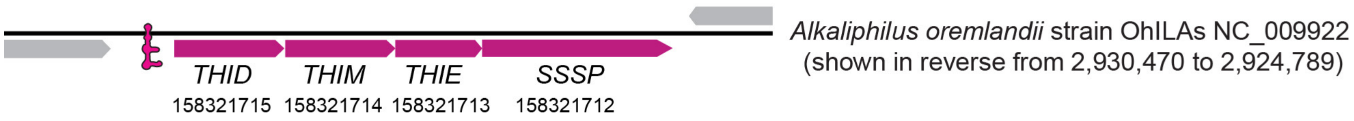

c

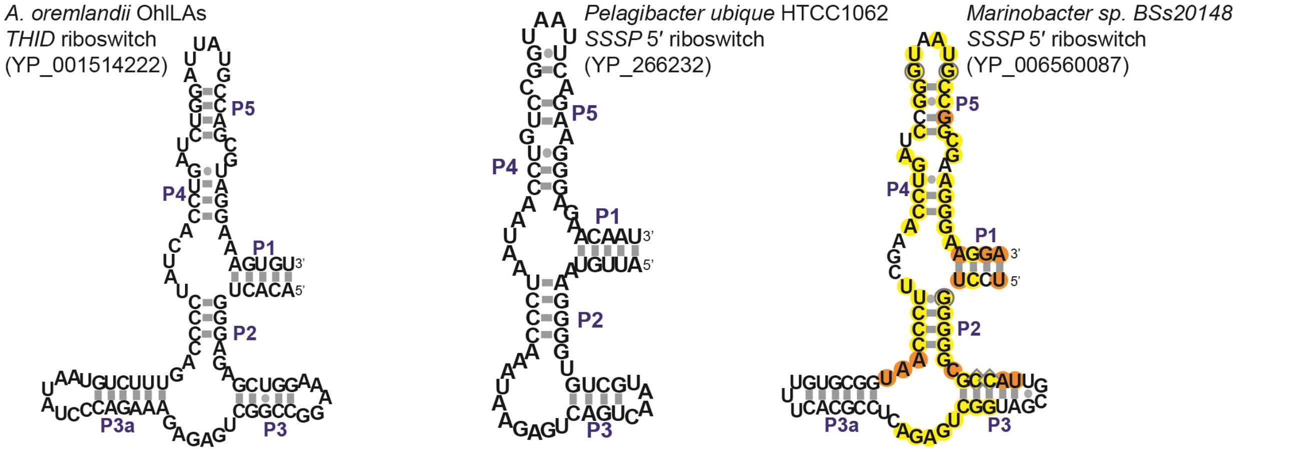

Supplementary Figure S6. Phylogenetic analysis of SSSP/SSSQ sequences retrieved in homolog searches. **(a)** This tree (the un-collapsed version of Fig. 5) used a greater number of amino acid positions from sequences in the bootstrap supported clade that contained all eukaryotic homologs as well as some bacterial and archaeal sequences in Supplementary Figure S5 (sequences between orange arrows). The extracted full-length sequences were re-aligned and a phylogeny constructed using RAxML with 100 bootstrap replicates. In addition, posterior probabilities were computed using MrBayes. Node support ≥70% and p ≥0.8 (grey circles) is shown and accession numbers are provided. Yellow icons indicate riboswitches resolved here (with details in Supplementary Table S2) and grey indicates the *Neurospora crassa* riboswitch (presumed present in other fungi as well and therefore shown at the species and lineage level), as well as a previously characterized *Micromonas* SSSP riboswitch. *V. carteri* and *C. reinhardtii* (3′ UTR) had elements that might be indicative of a riboswitch or riboswitch remnant. **(b)** The *Alkaliphilus oremlandii* strain OhlLAs operon containing classical thiamine pathway genes and *SSSP* (pink icon, Figure 5). Numbers below gene symbols are the Genbank Identifiers. **(c)** Predicted secondary structures of bacterial *SSSP* riboswitches. *Marinobacter* BSs20148 has many characteristics common in plant and algal riboswitches (coloring as in Fig. 2b) as does the *Achromobacter xylosoxidans* riboswitch (not shown).

Supplementary Table S1. Eukaryotic algae searched for novel genes. Genome browser URLs are provided for public JGI genomes without a publication. We also searched for novel gene homologs in genomes from *Phytophora sojae*, *P. ramorum*, *P. infestans*, *Aplanochytrium kerguelensis*, and *Perkinsus marinus*.

Lineage	Species	Genome Size (Mb)	Predicted Genes	Citation
GENOMES				
Chlorophyte	*Chlamydomonas reinhardtii*	121	15,143	(Merchant *et al.* 2007)
Chlorophyte	*Volvox carteri*	138	14,520	(Prochnik *et al.* 2010)
Cryptophyte	*Guillardia theta* CCMP2712	87	24,840	(Curtis *et al.* 2012)
Glaucophyte	*Cyanophora paradoxa*	70	27,921	(Price *et al.* 2012)
Haptophyta	*Emiliania huxleyi* CCMP1516	168	39,126	(Read *et al.* 2013)
Prasinophyte, Class II	*Bathycoccus prasinos* RCC1105	15	7,847	(Moreau *et al.* 2012)
Prasinophyte, Class II	*Micromonas* CCMP1545	22	10,575	(Worden *et al.* 2009)
Prasinophyte, Class II	*Micromonas* RCC299	21	10,056	(Worden et al 2009)
Prasinophyte, Class II	*Ostreococcus lucimarinus*	13	7,651	(Palenik *et al.* 2007)
Prasinophyte, Class II	*Ostreococcus* RCC809	13	7,492	http://genome.jgi-psf.org/OstRCC809_1/OstRCC809_1.home.html
Prasinophyte, Class II	*Ostreococcus tauri*	13	8,166	(Derelle *et al.* 2006)
Rhizarian	*Bigelowiella natans* CCMP2755	95	21,708	(Curtis et al 2012)
Rhodophyte	*Chondrus crispus*	105	9,606	(Collen *et al.* 2013)
Rhodophyte	*Cyanidioschyzon merolae*	17	5,331	(Matsuzaki *et al.* 2004)
Rhodophyte	*Pyropia yezoensis* U-51	43	10,327	(Nakamura *et al.* 2013)
Stramenopile	*Aureococcus anophagefferens* clone 1984	57	11,501	(Gobler *et al.* 2011)
Stramenopile	*Ectocarpus siliculosus*	196	16,256	(Cock *et al.* 2010)
Stramenopile	*Fragilariopsis cylindrus* CCMP1102	81	27,137	http://genome.jgi-psf.org/Fracy1/Fracy1.home.html
Stramenopile	*Phaeodactylum tricornutum* CCMP632	27	10,402	(Bowler *et al.* 2008)
Stramenopile	*Pseudo-nitzschia multiseries*	219	19,703	http://genome.jgi-psf.org/Psemu1/Psemu1.home.html
Stramenopile	*Thalassiosira pseudonana* CCMP1355	35	11,242	(Armbrust *et al.* 2004)
Trebouxiophyte	*Chlorella variabilis* NC64A	46	9,791	(Blanc *et al.* 2010)
Trebouxiophyte	*Coccomyxa subellipsoidea* C-169	49	9,851	(Blanc *et al.* 2012)
TRANSCRIPTOME ASSEMBLIES*			Contigs	
Prasinophyte, Class II	*Micromonas* CCMP2099	----	15,214	This publication†
Prasinophyte, Class II	*Micromonas* NEPCC29	----	8,892	This publication†
Prasinophyte, Class I	*Pyramimonas amylifera* CCMP720	----	20,736	This publication†
Prasinophyte, Class I	*Pyramimonas parkae* CCMP725	----	25,776	This publication†

For published genomes, genome size (rounded to mega base pairs, Mb) and number of predicted proteins are from the listed publication or the JGI Genome Portal (http://genome.jgi.doe.gov/). ---, not available. *Growth rates at the time of harvesting were 0.27 ± 0.06 d^{-1} (CCMP725), 0.21 ± 0.04 d^{-1} (CCMP720), 0.41 ± 0.09 d^{-1} (CCMP2099) and 0.46 ± 0.18 d^{-1} (NEPCC29). Transciptome contigs are available at http://camera.calit2.net/mmetsp/list.php.

Supplementary Table S2. Riboswitches identified here and reported in algae in previous publications. Note that although *THIC* homologs were present in glaucophytes and rhodophytes we did not detect riboswitches in these taxa (see Supplementary Table S1 for taxa investigated). Intron numbers are given for genes coming from genome (but not transcriptome) projects. For sequences generated herein (bold) transcriptome contigs numbers are as deposited in the CAMERA MMETSP database if not deposited in GenBank.

	Gene	Accession, JGI ID, or contig	Introns	Location	P3 (nt)	P3a (nt)	Citation
EUKARYOTES							
**Alveolate**							
Perkinsus marinus	*SSSP*	XP_002776900	0	5′ UTR	28	np	This study
Karenia brevis CCMP2229	*SSSP*	Contig 89418		5′ UTR	23	np	This study
**Chlorophyte**							
Chlamydomonas reinhardtii	*SSSF*	XP_001693504	7	5′ UTR	19	np	This study
Chlamydomonas reinhardtii	*THI4*	XP_001698672	7	5′ UTR			Croft et al.
Chlamydomonas reinhardtii	*THIC*	XP_001697756	~16	Mid-gene			Croft et al.
Volvox carteri	*SSSF*	XP_002949254	6	5′ UTR	44	np	This study
Volvox carteri	*THI4*	XP_002951140	7	5′ UTR			Croft et al.
Volvox carteri	*THIC*	XP_002953184	~16	Mid-gene			Croft et al.
**Cryptophyte**							
Guillardia theta CCMP2712	*SSSF*	EKX46008	3	5′ UTR	23	np	This study
Guillardia theta CCMP2712	*SSSQ*	EKX44698	3, 4	5′ UTR	23	np	This study
Guillardia theta CCMP2712	*THIM*	EKX55217	2	5′ UTR	22	np	This study
**Haptophyte**							
Emiliania huxleyi	*SSSP*	233786	3	3′ UTR	29	5	This study
**Prasinophyte, Class II**							
Micromonas CCMP1545	*SSSF*	XP_003062945	0	5′/3′ UTRs	23/24	np	structure†
Micromonas CCMP1545	*SSSP*	XP_003060673	0	5′/3′ UTRs	30/36	np	structure†
Micromonas CCMP2099	*ATS1*	**KF471078**		3′ UTR	115	np	This study
Micromonas RCC299	*ATS1*	XP_002504942	2	3′ UTR		np	Worden et al.
Micromonas RCC299	*~SSUA/THI5*	102273	0	3′ UTR	29	np	structure†
Micromonas RCC299	*UNK1*	XP_002500920	0	3′ UTR	23	np	structure†
Micromonas NEPCC29	*ATS1*	**Contig 1291**		3′ UTR	26	np	This study
Micromonas NEPCC29	*~SSUA/THI5*	**Contig 3874**		3′ UTR	id	np	This study
Micromonas NEPCC29	*UNK1*	**Contig 128**		3′ UTR	26	np	This study
Ostreococcus lucimarinus	*SSSF*	XP_001420601	0	5′ UTR			Worden et al.†
Ostreococcus lucimarinus	*SSSP*	XP_001417224	0	5′ UTR			Worden et al.†
Ostreococcus RCC809	*SSSF*	52484	0	5′ UTR	31	np	This study
Ostreococcus RCC809	*SSSP*	92066	0	5′ UTR	39	np	This study
Ostreococcus tauri	*SSSF*	24300, altered	0	5′ UTR			Worden et al.†
Ostreococcus tauri	*SSSP*	XP_003078733	0	5′ UTR			Worden et al.†
**Prasinophyte, Class I**							
Pyramimonas parkeae	*THIC*	**KF381171**		Mid-gene	39	np	This study
Pyramimonas amylifera	*THIC*	**Contig 11974**		Mid-gene	26	np	This study
**Rhizaria**							
Bigelowiella natans	*ATS1*	AY543013	2	3′	32	np	This study
**Stramenopile**							
Aplanochytrium kerguelensis	*SSSQ*	41763	1	3′ UTR	45	np	This study
Aureococcus anophagefferens	*SSSP*	61010	0	5′ UTR	16	np	This study

Aureococcus anophagefferens	*SSSP*	61385	0	5′ UTR	18	np	This study
Aureococcus anophagefferens	*~SSUA/THI5*	EGB02659	0	3′ UTR	16	np	This study
Fragilariopsis cylindrus	*SSSP*	193603	1	3′ UTR	7	40	This study
Fragilariopsis cylindrus	*THIC*	198330	0	3′ UTR	19	17	This study
Phaeodactylum tricornutum	*SSSP*	XP_002184897	1	3′ UTR	11	18	This study
Phaeodactylum tricornutum	*THIC*	XP_002182146	5	3′ UTR			Croft et al.
Pseudo-nitzschia multiseries	*SSSP*	238416	1	3′ UTR	7	40	This study
Thalassiosira pseudonana	*SSSP*	XP_002286623	3	3′ UTR	22	np	This study
Thalassiosira pseudonana	*THIC*	XP_002292310	2	3′ UTR			Croft et al.
Phytophthora infestans	*SSSP*	XP_002898660	2	5′ UTR	41	np	This study
Phytophthora ramorum	*SSSP*	79316	4	5′ UTR	41	np	This study
Phytophthora sojae	*SSSP*	EGZ18412*	2	5′ UTR	41	np	This study
BACTERIA							
Achromobacter xylosoxidans A8	*SSSP*	YP_003978618	0	5′	13	14	This study
Alkaliphilus oremlandii OhILAs	*THID*	YP_001514222	0	5′	17	20	This study
Halomonas elongata DSM 2581	*SSSP*	YP_003896589	0	5′	18	13	This study
Halomonas titanicae BH1	*SSSP*	ZP_21729617	0	5′	17	12	This study
Leptolyngbya PCC6406	*SSSP*	ZP_21047934	0	5′	16	np	This study
Marinobacter ELB17	*SSSP*	ZP_01735446	0	5′	14	15	This study
Marinobacter BSs20148	*SSSP*	YP_006560087	0	5′	14	15	This study
Pelagibacter ubique HTCC1062	*SSSP*	YP_266232	0	5′	12	np	This study[†]

Length of P3 stems and the much rarer P3a stems is listed in nucleotides (nt); np, not present; id, incomplete data (riboswitch structure was incomplete due to transcriptome contig termination; Supplementary Fig. S1b). *Riboswitches with similar structure were observed in *P. sojae SSSP* homologs EGZ18412 and EGZ29809; [†]Presence of these riboswitches was proposed in Worden et al. 2009, but structures were not resolved and are provided here for all but *O. lucimarinus* and *O. tauri*. Croft et al. refers to (Croft *et al.* 2007) and Worden et al. refers to (Worden et al 2009). *~SSUA/THI5* indicates *SSUA/THI5*-like sequences encoding proteins in PF09084. Note that in *Micromonas* CCMP2099 the *SSUA/THI5*-like RNA-seq contig terminated before the UTR, hence for this sequence and four others indicated in Fig. 4a we could not determine whether a riboswitch was present.

		HMP-P Key Enzymes		HET-P Key Enzymes		THI6 domains	THI6 domains / TH1 domains	TH1 domains	Arrangement		Possibly B_1 related		
Lineage	Species	THI5 (PF09084)	THIC (PF01964)	THI4 (PF01946)	THIG (PF05690)	THIM (PF02110)	THIE (PF02581)	THID (PF08543)	TH1	THI6	TPK1 (PF04263, PF04265)	TENA (PF03070)	SSUA/ THI5-like (PF09084)
MODEL ORGANISMS													
Fungi	*N. crassa*	XP_001728011	---	XP_959972	---	XP_001728496	XP_001728496	XP_963110.2	---	✓	XP_959392	XP_963110.2	---
Fungi	*S. cerevisiae*	NP_116597	---	NP_011660	---	NP_015110	NP_015110	NP_014586	---	✓	NP_014786	NP_014586	---
L.Plants	*A. thaliana*	---	NP_180524	NP_200288	---	NP_189045	NP_173707.2	NP_173707.2	✓	---	NP_566026	---	---
L.Plants	*O. sativa*	---	NP_001050897	NP_001059841	---	BAD52630	ABG21903	ABG21903	✓	---	NP_001055337	Os03g19390	---
L.Plants	*P. patens*	---	XP_001766848	XP_001753698	---	XP_001786765	XP_001773462	XP_001773462	✓	---	XP_001769544	XP_001769831	XP_001762425
L.Plants	*S. moellendorffii*	---	XP_002984674	XP_002973063	---	XP_002972200	XP_002991097	XP_002991097	✓	---	XP_002968411	XP_002979391	148807
ALGAL GENOMES			---										
Chloro.	*C. reinhardtii*	---	XP_001697756	XP_001698672	---	XP_001696630	Cre08.g365600	Cre08.g365600	✓	---	XP_001690140	---	XP_001690423
Chloro.	*V. carteri*	---	XP_002953184	XP_002951140	---	ADI46896	XP_002948683	XP_002948683	✓	---	XP_002954012	XP_002948683	XP_002956902
Crypto.	*G. theta*	---	---	162720	---	62773	62657	62657	✓	---	139437	60307	165857
Glauco.	*C. paradoxa*	---	contig 7793	contig 38274	---	contig 39106	---	---	---	---	contig 6654	---	---
Hapto.	*E. huxleyi*	---	---	---	cp,YP_277380	---	102278	102278	✓	---	234208, 56122	---	98742, 113484
Prasino.	*B. prasinos*	---	---	---	---	---	---	---	---	---	---	---	---
Prasino.	*M.* sp. CCMP1545	---	---	---	---	XP_003063149	XP_003062944	XP_003062944	✓	---	XP_003063033	XP_003063148	XP_003058640
Prasino.	*M.* sp. RCC299	---	---	---	---	---	---	XP_002502192	---	---	XP_002508744	---	XP_002504260
Prasino.	*O. lucimarinus*	---	---	---	---	XP_001420768	XP_001420600	XP_001420600	✓	---	XP_001415504	XP_001420767	---
Prasino	*O.* RCC809	---	---	---	---	59648	59643	59643	✓	---	24781	59644	---
Prasino.	*O. tauri*	---	---	---	---	XP_003082249	XP_003082241	XP_003082241	✓	---	10431	XP_003082247	---
Rhiz.	*B. natans*	---	---	---	---	147452	28959	28959	✓	---	129605	84573	88583
Rhodo.	*C. crispus*	---	CDF41045.1	---	cp, YP_007627318	---	---	---	---	---	---	---	---
Rhodo.	*C. merolae*	---	BAM79672	---	cp, NP_848998	---	BAM81836	BAM81496	---	---	BAM79721	---	---
Rhodo.	*P. yezoensis*	---	contig 5158	---	cp, YP_537042	---	---	contigs 7296, 30437	---	---	---	---	---
Strameno.	*A. anophagefferens*	---	---	---	EGB11989	---	---	EGB06309	---	---	EGB11871	EGB02605	EGB02659
Strameno.	*F. cylindrus*	---	225659	---	cp, scf_95:27066-27872	---	181136	181136	✓	---	142551	199067	252997
Strameno.	*P. tricornutum*	---	XP_002182146	---	cp, YP_874486	---	XP_002181508	XP_002181508	✓	---	XP_002180581	XP_002179469	XP_002186483
Strameno.	*P. multiseries*	---	255053	---	cp, scf187	---	320126	320126	✓	---	264355	228645	318043
Strameno.	*T. pseudonana*	---	XP_002292310	---	cp, YP_874615	---	6388	6388	✓	---	255560	XP_002289152	XP_002295704
Treboux.	*C. variabilis*	---	EFN53744	EFN55828	---	EFN53626	EFN53733	EFN53733	✓	---	EFN53214	EFN53733	EFN57875
Treboux.	*C. subellipsoidea*	---	EIE20848.1	EIE24385.1	---	EIE20254.1	EIE20255.1	EIE20255.1	✓	---	EIE21631.1	---	XP_001690423.1
TRANSCRIPTOME ASSEMBLIES													
Prasino.	*M.* CCMP2099	---	nf	nf	Nf	nf	nf	KF381168	---	---	nf	nf	KF381167
Prasino.	*M.* NEPCC29	---	nf	nf	Nf	nf	nf	nf	---	---	14507	nf	3874
Prasino.	*P. amylifera*	---	11974	121	Nf	nf	5338	5338	✓	---	39566	nf	95385
Prasino.	*P. parkeae*	---	KF381171	nf	Nf	KF381172	KF381169	KF381169	reverse order	---	nf	nf	KF381170

Supplementary Table S3. Summary of thiamine related genes recovered from genomes and transcriptomes. Arrangement columns indicate whether a TH1 fusion of THID and THIE is present or a THI6 fusion of THIE and THIM. Note that *E. huxleyi* has a thiamine pathway with all necessary enzymes except THIC, and has algal SSUA/THI5-like but cannot synthesize thiamine *de novo*, making it unlikely algal SSUA/THI5-like proteins (3 of which had affiliated riboswitches) are directly involved in HMP-P synthesis. GenBank accessions, JGI protein IDs or contig numbers are provided from publically available genome projects. Lineage abbreviations: L. Plants: Land Plant, Chloro: Chlorophyte, Crypto: Cryptophyte, Glauco: Glaucophytes, Prasino: Prasinophyte, Rhiz: Rhizarian, Rhodo: Rhodophyte, Strameno: Stramenopile, Treboux.: Trebouxiophyceae. --- denotes genes not found in sequenced genomes, nf denotes genes not found in transcriptomes, cp denotes genes in the chloroplast genome.

Supplementary Table S4. qPCR primers and probes developed or used herein. A 6-FAM reporter dye and Black Hole Quencher 1 (or 2) were used at the probe 5′ and 3′ termini, respectively, except for the RCC299 Actin probe. The latter used a Minor Groove Binding quencher at the 3′ terminus.

Primer	Protein ID	Sequence (5'– 3')	Amplicon Length	Efficiency (%)
Micromonas CCMP1545				
Actin F	48012	GGACTTTGAGAACGAGATGCAA	98	100
Actin R	48012	GCTCGTCCGAGATGGTGATC		
Actin P	48012	CGAGCTCCCCGACGGTCAGG		
Beta-tubulin F	70188	GCCACGGCCGCTATCTC	67	99
Beta-tubulin R	70188	TCATCGACTTCTTTCGTGGACAT		
Beta-tubulin P	70188	CCGCTTCAGCCCTTTTCCGTGG		
SSUA/THI5-like *F*	58387	CTCGAGGGAATCGACCTCAA	76	99
SSUA/THI5-like *R*	58387	CGCACAGACACGGGTTGTAC		
SSUA/THI5-like *P*	58387	TCTTCACCCTGCTCGATAACGGCG		
SSSF F	48760	CACGTCACCGCGCATCT	79	105
SSSF R	48760	ACTGCGAGACTTGGTACGTGAA		
SSSF P	48760	CTCCGAATCATCACCTACGGCTGCA		
SSSP F	60112	GTCACGCCGTCCGAGATG	97	106
SSSP R	60112	AGAGCTCGGCGGAGATGA		
SSSP P	60112	TCGTCATGGTCACGCTCATCATCG		
Micromonas RCC299				
*Actin F**	90942	GCCCTCGTGTGCGATAAC	89	100
*Actin R**	90942	CCGACGATGGAGGGAAAGAC		
*Actin P**	90942	CCGGCCTTGACCATGC		
*Beta-tubulin F**	104730	TTCCCCCGAAGGGTCTGA	60	95, 104
*Beta-tubulin R**	104730	CCTGAACAGCGGTGGTGTT		
*Beta-tubulin P**	104730	TGGCGGCCACCTTCATCGG		
UNK1 F	56895	GGCAATCGTTGCGAGGAAT	103	109
UNK1 R	56895	ACGGCGTGTTGGTGTTGTT		
UNK1 P	56895	CCGGACAACTGCGTCATCCCCA		
ATS1 F	106264	GAACTCCGGCATATCCGAAA	73	106
ATS1 R	106264	GGTCTTGGCGGTGTTGAGA		
ATS1 P	106264	AGCTCCCGCGGTACCCTCATCTG		
SSUA/THI5-like *F*	102273	CCTCAGAGACCGCAACTCATC	72	105
SSUA/THI5-like *R*	102273	TCGAGGTCCTTCATGGAGTTTATC		
SSUA/THI5-like *P*	102273	CCATCGTGGCTCTCAAGTCCTCCG		

Primer efficiency was determined using plasmid standard curves (10^8 to 10^1 copies rxn^{-1}, in 10-fold increments). Efficiencies were calculated as $(10^{(-1/m)}-1)$, where *m* is the slope of the regression line F: Forward primer, R: reverse primer, P: probe. *Previously published *Actin* primers (McDonald *et al.* 2010) and *Beta-tubulin* primers (Cuvelier 2010).

Supplementary Table S5. *Micromonas* RCC299 gene expression responses relative to positive controls for thiamine (Thi) manipulations and precursor additions. A subset of the below data is provided in Fig. 3d. QPCR technical triplicates were performed in addition to the biological replication.

Treatment	Gene	4 hrs	Day 1	Day 2	Day 3	Day 4	Day 5
+10 µM Thi	*UNK1*	---	---	---	---	---	---
No Thi	*UNK1*	---	---	---	---	4.38 (1.57,1.15)	4.71 (0.73,0.64)
No Thi, +HET	*UNK1*	3.54 (0.20,0.19)	3.74 (0.15,0.14)	3.47 (0.20,0.19)	2.80 (0.24,0.22)	4.78 (0.64,0.56)	11.95 (4.71,3.38)
No Thi, +HMP	*UNK1*	4.83 (0.23,0.22)	3.85 (0.48,0.43)	3.90 (0.20,0.19)	3.54 (0.35,0.31)	5.53 (0.32,0.31)	16.42 (1.15,1.08)
No Thi,+HET+HMP	*UNK1*	2.47 (0.40,0.34)	3.30 (0.17,0.16)	4.65 (0.99,0.82)	3.41 (0.19,0.18)	7.00 (2.11,1.62)	16.22 (4.08,3.26)
+10 µM Thi	*ATS1*	---	---	---	---	---	---
No Thi	*ATS1*	---	2.90 (1.11,0.80)	5.89 (2.92,1.95)	9.36 (0.16,0.16)	24.96 (5.57,4.55)	61.47 (11.16,9.45)
No Thi, +HET	*ATS1*	4.02 (0.58,0.51)	5.89 (0.14,0.13)	11.57 (0.23,0.22)	13.15 (1.90,1.66)	26.25 (2.63,2.39)	105.11 (11.37,10.26)
No Thi, +HMP	*ATS1*	6.57 (1.40,1.16)	4.06 (0.37,0.34)	11.83 (0.61,0.58)	15.73 (0.55,0.53)	33.59 (3.11,2.84)	137.76 (8.59,8.08)
No Thi,+HET+HMP	*ATS1*	3.46 (0.72,0.60)	3.97 (0.33,0.30)	12.23 (0.72,0.68)	17.22 (0.74,0.71)	42.84 (6.03,5.29)	131.08 (28.18,23.20)
+10 µM Thi	*SSUA/THI5* -like	---	---	---	---	---	---
No Thi	As above	---	3.32 (3.35, 1.67)	---		3.25 (1.88, 1.19)	---
No Thi, +HET	As above	---	7.22 (0.23, 0.22)	2.09 (0.26,0.23)	---	---	2.72 (0.96, 0.71)
No Thi, +HMP	As above	---	9.14 (0.25, 0.24)	---	---	---	4.24 (0.96, 0.78)
No Thi,+HET+HMP	As above	---	8.37 (0.53, 0.49)	---	---	---	2.19 (0.56, 0.44)

Micromonas RCC299 fold changes were calculated using *Beta-tubulin* as the housekeeper and the control treatments as the calibrator. Fold changes are listed followed by positive SD and negative SD. Fold changes <2 are not shown, indicated by ---. HET and HMP were added at a final concentration of 10 µM. Generations achieved during each treatment are as follows: Day 1 (20 hrs): 1.15-1.52 generations, Day 2 (44 hrs): 2.50-2.84 generations, Day 3 (68 hrs): 2.82-3.29 generations, Day 4 (92 hrs): 2.95-3.71 generations, Day 5 (116 hrs): 2.95-4.09 generations. HMP: 4-amino-5-hydroxymethyl-2-methyl-pyrimidine, HET: 4-methyl-5-β-hydroxyethylthiazole.

Supplementary Table S6a. *Micromonas* CCMP1545 gene expression responses relative to positive controls in thiamine (Thi) manipulations and precursor moiety additions for transfer experiment (Fig. 3a, c). QPCR technical triplicates were performed in addition to the biological replication.

Treatment	Gene	Day 3	Day 6	Day 8
+1 µM Thi	*SSUA/THI5*-like	---	2.22 (0.06,0.06)	---
No Thi	*SSUA/THI5*-like	---	---	---
No Thi, +HET	*SSUA/THI5*-like	---	---	---
No Thi, +HMP	*SSUA/THI5*-like	---	---	---
No Thi,+HET,+HMP	*SSUA/THI5*-like	---	---	n/a
+1 µM Thi	*SSSF*	---	---	---
No Thi	*SSSF*	14.85 (0.38,0.37)	11.54 (1.79,1.55)	6.54 (0.68,0.61)
No Thi, +HET	*SSSF*	12.44 (6.36,4.21)	11.81 (3.15,2.48)	6.36 (0.75,0.67)
No Thi, +HMP	*SSSF*	18.00 (2.14,1.91)	17.60	9.32 (0.91,0.83)
No Thi,+HET+HMP	*SSSF*	9.82 (3.31,2.48)	15.98 (2.88,2.44)	n/a
+1 µM Thi	*SSSP*	---	---	---
No Thi	*SSSP*	13.45 (0.40,0.39)	17.23 (0.61,0.59)	7.33 (0.19,0.19)
No Thi, +HET	*SSSP*	9.20 (3.81,2.69)	13.82 (5.95,4.16)	6.83 (1.16,0.99)
No Thi, +HMP	*SSSP*	13.35 (4.12,3.15)	18.24 (8.24,5.67)	9.65 (0.64,0.60)
No Thi,+HET+HMP	*SSSP*	8.59 (1.58,1.33)	18.76 (1.38,1.29)	n/a

Fold changes were calculated using *Beta-tubulin* as the housekeeper and controls as the calibrator. Fold changes are listed followed by positive SD and negative SD. Fold changes <2 are not shown, indicated by ---. HET and HMP were added at a final concentration of 1 µM. Generations achieved during each treatment are as follows: Day 3 (69 hrs): 2.09-2.41 generations, Day 6 (141 hrs): 4.24-5.97 generations, Day 8 (192 hrs): 4.68- 8.25 generations. HMP: 4-amino-5-hydroxymethyl-2-methyl-pyrimidine, HET: 4-methyl-5-β-hydroxyethylthiazole. n/a, not available.

Supplementary Table S6b. *Micromonas* CCMP1545 gene expression responses to thiamine (Thi) manipulations in the preliminary CCMP1545 transfer experiments. This was performed with biological quadruplicates but precursors were not tested (growth rates not shown). QPCR technical triplicates were performed in addition to the biological replication.

Treatment	Gene	Day 2	Day 5
+1 µM Thi	*SSUA/THI5*-like	---	---
No Thi	*SSUA/THI5*-like	2.19 (0.53, 0.43)	---
+1 µM Thi	*SSSF*	---	---
No Thi	*SSSF*	14.69 (1.72, 1.54)	17.24 (1.23, 1.15)
+1 µM Thi	*SSSP*	---	---
No Thi	*SSSP*	10.70 (2.34, 1.92)	11.28 (2.18, 1.82)

Fold changes were calculated using *Beta-tubulin* as the housekeeper and controls as the calibrator. Fold changes are listed followed by positive SD and negative SD. Fold changes <2 are not shown, indicated by ---. HET and HMP were added at a final concentration of 1 µM. Total generations achieved are as follows: Day 2 (45 hrs): 1.54-19.2 generations, Day 5 (117 hrs): 3.21-4.00 generations.

References

Armbrust EV, Berges JA, Bowler C, Green BR, Martinez D, Putnam NH *et al.* (2004). The genome of the diatom *Thalassiosira pseudonana*: ecology, evolution, and metabolism. *Science* 306: 79-86.

Blanc G, Duncan G, Agarkova I, Borodovsky M, Gurnon J, Kuo A *et al.* (2010). The *Chlorella variabilis* NC64A genome reveals adaptation to photosymbiosis, coevolution with viruses, and cryptic sex. *Plant Cell* 22: 2943-2955.

Blanc G, Agarkova I, Grimwood J, Kuo A, Brueggeman A, Dunigan DD *et al.* (2012). The genome of the polar eukaryotic microalga *Coccomyxa subellipsoidea* reveals traits of cold adaptation. *Genome Biol* 13: R39.

Bowler C, Allen AE, Badger JH, Grimwood J, Jabbari K, Kuo A *et al.* (2008). The *Phaeodactylum* genome reveals the evolutionary history of diatom genomes. *Nature* 456: 239-244.

Cock JM, Sterck L, Rouze P, Scornet D, Allen AE, Amoutzias G *et al.* (2010). The *Ectocarpus* genome and the independent evolution of multicellularity in the brown algae. *Nature* 465: 617-621.

Collen J, Porcel B, Carre W, Ball SG, Chaparro C, Tonon T *et al.* (2013). Genome structure and metabolic features in the red seaweed *Chondrus crispus* shed light on evolution of the Archaeplastida. *Proc Natl Acad Sci U S A* 110: 5247-5252.

Croft MT, Moulin M, Webb ME, Smith AG (2007). Thiamine biosynthesis in algae is regulated by riboswitches. *Proc Natl Acad Sci U S A* 104: 20770-20775.

Curtis BA, Tanifuji G, Burki F, Gruber A, Irimia M, Maruyama S *et al.* (2012). Algal genomes reveal evolutionary mosaicism and the fate of nucleomorphs. *Nature* 492: 59-65.

Cuvelier ML (2010). New insights into the diversity, distribution and ecophysiology of marine picoeukaryotes. PhD thesis, University of Miami.

Derelle E, Ferraz C, Rombauts S, Rouze P, Worden AZ, Robbens S *et al.* (2006). From the Cover: Genome analysis of the smallest free-living eukaryote *Ostreococcus tauri* unveils many unique features. *Proc Natl Acad Sci U S A* 103: 11647-11652.

Gobler CJ, Berry DL, Dyhrman ST, Wilhelm SW, Salamov A, Lobanov AV *et al.* (2011). Niche of harmful alga *Aureococcus anophagefferens* revealed through ecogenomics. *Proc Natl Acad Sci U S A* 108: 4352-4357.

Matsuzaki M, Misumi O, Shin IT, Maruyama S, Takahara M, Miyagishima SY *et al.* (2004). Genome sequence of the ultrasmall unicellular red alga Cyanidioschyzon merolae 10D. *Nature* 428: 653-657.

McDonald SM, Plant JN, Worden AZ (2010). The mixed lineage nature of nitrogen transport and assimilation in marine eukaryotic phytoplankton: a case study of *Micromonas*. *Mol Biol Evol* 27 2268-2283.

Merchant SS, Prochnik SE, Vallon O, Harris EH, Karpowicz SJ, Witman GB *et al.* (2007). The *Chlamydomonas* genome reveals the evolution of key animal and plant functions. *Science* 318: 245-250.

Moreau H, Verhelst B, Couloux A, Derelle E, Rombauts S, Grimsley N *et al.* (2012). Gene functionalities and genome structure in *Bathycoccus prasinos* reflect cellular specializations at the base of the green lineage. *Genome Biol* 13: R74.

Nakamura Y, Sasaki N, Kobayashi M, Ojima N, Yasuike M, Shigenobu Y *et al.* (2013). The first symbiont-free genome sequence of marine red alga, Susabi-nori (*Pyropia yezoensis*). *PLoS One* 8: e57122.

Palenik B, Grimwood J, Aerts A, Rouze P, Salamov A, Putnam N *et al.* (2007). The tiny eukaryote *Ostreococcus* provides genomic insights into the paradox of plankton speciation. *Proc Natl Acad Sci U S A* 104: 7705-7710.

Price DC, Chan CX, Yoon HS, Yang EC, Qiu H, Weber APM *et al.* (2012). *Cyanophora paradoxa* genome elucidates origin of photosynthesis in algae and plants. *Science* 335: 843-847.

Prochnik SE, Umen J, Nedelcu AM, Hallmann A, Miller SM, Nishii I *et al.* (2010). Genomic analysis of organismal complexity in the multicellular green alga *Volvox carteri*. *Science* 329: 223-226.

Read BA, Kegel J, Klute MJ, Kuo A, Lefebvre SC, Maumus F *et al.* (2013). Pan genome of the phytoplankton *Emiliania* underpins its global distribution. *Nature* 499: 209–213.

Wachter A, Tunc-Ozdemir M, Grove BC, Green PJ, Shintani DK, Breaker RR (2007). Riboswitch control of gene expression in plants by splicing and alternative 3' end processing of mRNAs. *Plant Cell* 19: 3437-3450.

Worden AZ, Lee JH, Mock T, Rouze P, Simmons MP, Aerts AL *et al.* (2009). Green evolution and dynamic adaptations revealed by genomes of the marine picoeukaryotes *Micromonas*. *Science* 324: 268-272.
